# Acoustic Streaming‐Induced Multimodal Locomotion of Bubble‐Based Microrobots

**DOI:** 10.1002/advs.202304233

**Published:** 2023-10-26

**Authors:** Nima Mahkam, Amirreza Aghakhani, Devin Sheehan, Gaurav Gardi, Robert Katzschmann, Metin Sitti

**Affiliations:** ^1^ Physical Intelligence Department Max Planck Institute for Intelligent Systems 70569 Stuttgart Germany; ^2^ Institute for Biomedical Engineering ETH Zurich Zurich 8092 Switzerland; ^3^ Institute of Biomaterials and Biomolecular Systems University of Stuttgart 70569 Stuttgart Germany; ^4^ Department of Mechanical and Process Engineering ETH Zurich Zurich 8092 Switzerland; ^5^ School of Medicine Koç University Istanbul 34450 Turkey; ^6^ College of Engineering Koç University Istanbul 34450 Turkey

**Keywords:** acoustic microstreaming, acoustic‐powered microrobots, biomedical robots, bubble‐based propulsion, multimodal locomotion

## Abstract

Acoustically‐driven bubbles at the micron scale can generate strong microstreaming flows in its surrounding fluidic medium. The tunable acoustic streaming strength of oscillating microbubbles and the diversity of the generated flow patterns enable the design of fast‐moving microrobots with multimodal locomotion suitable for biomedical applications. The acoustic microrobots holding two coupled microbubbles inside a rigid body are presented; trapped bubbles inside the L‐shaped structure with different orifices generate various streaming flows, thus allowing multiple degrees of freedom in locomotion. The streaming pattern and mean streaming speed depend on the intensity and frequency of the acoustic wave, which can trigger four dominant locomotion modes in the microrobot, denoted as translational and rotational, spinning, rotational, and translational modes. Next, the effect of various geometrical and actuation parameters on the control and navigation of the microrobot is investigated. Furthermore, the surface‐slipping multimodal locomotion, flow mixing, particle manipulation capabilities, the effective interaction of high flow rates with cells, and subsequent cancerous cell lysing abilities of the proposed microrobot are demonstrated. Overall, these results introduce a design toolbox for the next generation of acoustic microrobots with higher degrees of freedom with multimodal locomotion in biomedical applications.

## Graphical abstract

Addressing microrobots' limited maneuverability; the acoustically‐powered micron‐scale robots with microorganism‐inspired motions are developed. These robots house two coupled microbubbles that create complex acoustic‐streaming yielding various flow patterns and allowing the microrobots to move swiftly. These microrobots have proven to excel in multimodal locomotion, flow mixing, and cell lysing, making them ideal for diverse biomedical uses.

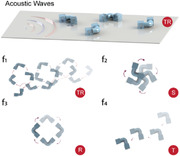

## 1 Introduction

Multimodal locomotion of organisms can enable adaptive behaviors in diverse classes of tasks. Behavioral responses range from flipping, spinning, coiling, lateral writhing, and rolling in large animals like salamanders^[^ ^1^ ^]^ and namib^[^ ^2^ ^]^ to twisting, rolling, tumbling, and symmetric and asymmetric flagella undulations in small structures, such as red blood cells^[^ ^3^ ^]^ and human sperms.^[^ ^4^ ^]^ While cell‐sized entities use a precise sequence of biochemical and morphological environmental changes to respond and switch their mode of locomotion, larger creatures use their neural control system (i.e., brain) to initiate an active response or change their operating gait. Alternatively, synthetic microrobots rely on external control inputs to alter their behavior and manipulate the surrounding environment. This external control system links the scalability and microenvironment adaptation of small entities with the precise controllability of larger creatures and enables multimodal locomotion on the microscale. The high mobility of microrobots with multiple degrees of freedom (DOF) would enable targeted drug delivery and minimally invasive medical applications^[^ ^5^ ^]^ inside the deep, hard‐to‐reach, and confined human body sites with varying complex morphologies.

Microrobots have attracted much attention recently due to the rapid progress in various micro/nanofabrication techniques and their promising applications in the environmental and biomedical fields.^[^ ^6^ ^]^ External actuation,^[^ ^7^, ^8^, ^9^ ^]^ self‐propulsion,^[^ ^10^, ^11^ ^]^ and biohybrid control^[^ ^12^ ^]^ of such micron‐sized systems have been investigated to locomote these small‐scale systems in given complex real‐world environments. A large group of externally actuated microrobots relies on magnetic,^[^ ^13^ ^]^ optical,^[^ ^14^ ^]^ electrical,^[^ ^15^ ^]^ or acoustic fields^[^ ^16^ ^]^ to harvest energy and propel. Rolling,^[^ ^17^, ^18^ ^]^ non‐reciprocal swimming,^[^ ^19^ ^]^ or rectilinear sliding^[^ ^20^ ^]^ are examples of main locomotion modes that recent works on small‐scale robotics have shown.^[^ ^21^ ^]^ Although untethered soft millirobots with high degrees of freedom and multimodal locomotion have been realized by the interaction of an elastomer body with an external magnetic field,^[^ ^22^ ^]^ existing micron‐size robots have limited mobility and are mainly restricted with a single DOF in motion, which narrows their presence to only lab‐controlled conditions. Among different actuation techniques, acoustic manipulation has emerged as a biocompatible method overcoming the constraints of the other actuation modalities, e.g., limited penetration depth, specific material or medium dependence, or toxicity of the integrated materials. Acoustic manipulation of microrobots represents a precise manipulation tool for various research fields in biology and engineering to study single‐cell morphogenesis^[^ ^23^ ^]^ and to drive microrobots inside a complex medium.^[^ ^24^ ^]^ The working principle of acoustic manipulation is to convert the distributed high‐intensity‐pressure waves into strong propulsion forces capable of powering small‐scale robots.^[^ ^25^ ^]^

One effective propulsion method is the acoustic streaming of an excited (e.g., resonating) microbubble within the path of a sound wave to generate a net thrust for a micro/millimeter‐sized robot.^[^ ^26^ ^]^ Flexibility in shape, size, and versatility in generated forces make bubble‐based propulsion a strong candidate for microrobotic applications. Generally, for microstructures operating with bubble oscillation, the bubble does not form the microrobot's main body; however, it plays a vital role in generating the thrust force. At the micron scale, 3D nanoprinting using two‐photon lithography has enabled the design of complex 3D microstructures with cavities for trapping a microbubble^[^ ^27^, ^28^ ^]^ that can drive the microrobot under an oscillating pressure field. Symmetry‐breaking methods around the microrobot have produced a unidirectional net moment and a rectilinear motion by integrating a fin‐shaped microstructure into the microrobot^[^ ^29^ ^]^ or by magnetically tilting the microrobot's body.^[^ ^30^ ^]^ Using similar concepts, Liu et al. demonstrated the potential of using multiple different‐sized bubbles–having different resonance frequencies–to generate independent thrusts in three different directions for a millimeter‐size drone.^[^ ^31^ ^]^ Multi‐bubble‐based propulsion was also used to actuate two distinct magneto‐acoustic micro‐propellers with two propeller designs and different bubble arrangements to separately achieve rotational or spiral modes.^[^ ^32^ ^]^ Similarly, the use of multiple bubbles for selective actuation^[^ ^33^ ^]^ was proposed to generate different locomotion types; where various locomotion modes were achieved by utilizing bubbles of various sizes at different designs. However, using trapped cylindrical bubbles substantially decreases the bubble stability, and using multiple bubbles dramatically increases the microrobot's size. Moreover, the existing robots are limited to a simple locomotion type and incapable of generating multimodal locomotion, where different locomotion modes require distinct microrobot designs. Such limitations require alternative approaches to design and operate multi‐DOF microrobots for better adaptability in diverse environments and medical tasks.

Besides locomotion, the streaming flow generated by a vibrating microbubble can also be used for particle trapping, particle transport, pattern formation in 3D, and fluid mixing in fundamental and biomedical research.^[^ ^34^, ^35^ ^]^ Such delicate functions harness the strong streaming flows inside microfluidic devices that offer flexible spatial and temporal control over fluid movement.^[^ ^36^ ^]^ Oscillatory bubbles have the potential to provide streamlines satisfying the need for such applications.^[^ ^37^ ^]^ The microstreaming of an oscillatory bubble offers a high degree of spatial control over individuals and groups of cells. Researchers have used bubble arrays excited with a low‐amplitude acoustic wave to move and rotate single cells for observation and analysis.^[^ ^35^ ^]^ Similarly, the streaming interaction of two oscillating bubbles has been used for size‐based separation and pumping simultaneously. Size‐based particle separation was achievable down to a 5 µm difference in the diameter using such bubble‐generated microstreaming.^[^ ^38^ ^]^

Here, we present a microbubble propulsion mechanism that uses several flow patterns around the microrobot to perform different locomotion types in a single microrobot design. To enable multimodal locomotion, we introduce two coupled similar‐sized spherical microbubbles, which biases the bubble‐induced streaming pattern and hence the locomotion mode. Two different‐sized orifices (i.e., diameter variations of 2 µm) are integrated into a single cavity, making it possible to switch the effective contribution of an individual orifice on the overall flow based on the wave's pressure and frequency. Additionally, the interactions of two bubbles with similar‐sized orifices, but different arrangements make it possible to increase the diversity of the flow patterns achievable with an acoustically‐powered microrobot. This amplitude and frequency dependence of the microstreams of two orifices on a cavity changes the amplitude and direction of the forces acting on the microrobot and hence its behavior. The extra degrees of freedom are added to the microrobots by simply tuning the interaction of the orifices and the streams around the microstructure. Additionally, to better visualize the effect of orifice size and its spatial position, we compare the locomotion behavior of three microrobotic designs with a varying number and arrangement of orifices. Next, we analyze the effect of nozzles on the cavity and their coupled effect on the microstreaming under a vast range of acoustic inputs. Finally, we demonstrate the cargo trapping, transport, cell‐lysing, and mixing functionalities of the microrobot under the excitement of different amplitudes and frequencies of the applied acoustic waves.

## 2 Microrobot Design and Fabrication

**Figure 1:**
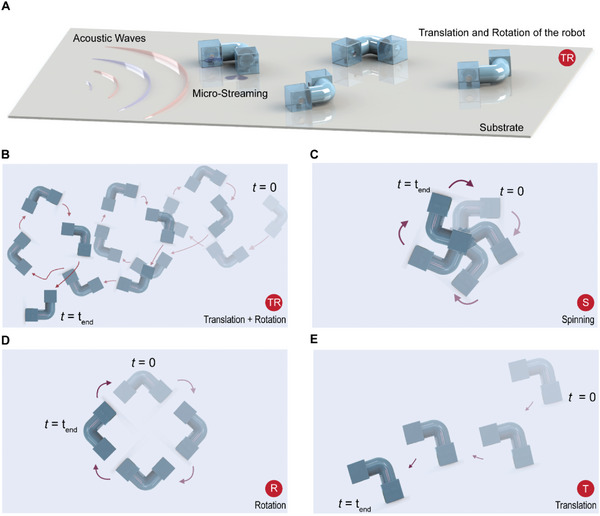
Locomotion modes of the L‐shaped acoustically‐powered microrobot. A) Schematic of the microrobot propulsion due to acoustic streaming; the robot exhibits a certain locomotion mode selected by the sound wave of a certain excitation intensity and frequency. Four locomotion modes appear due to the symmetric and asymmetric microstreaming patterns generated by the coupled oscillating bubbles. B) Translation and rotation –TR– mode makes the microrobot follow a spiral trajectory. C) Spinning –S– mode lets the microrobot spin around an origin point on the body. D) Rotation –R– mode causes the microrobot to rotate around an external origin point. E) Translation –T– mode moves the microrobot on a line trajectory.

An L‐shaped microrobot is designed with tunable locomotion modes, as presented in **Figure** **1**. The microrobot shows four different locomotion modes with tunable properties excited at a different acoustic frequency or amplitude. The microrobot possesses two similar‐sized cavities (air‐filled bubbles) with multiple orifices. Tuning the acoustic frequency and amplitude alternates the steady flow patterns around the microrobot. These different fluid flows result in four tunable locomotion modes: i) translational and rotational mode (TR)–microrobot follows a spiral trajectory as in Figure 1B, ii) spinning mode (S)–microrobot spins around a body‐centered point as in Figure 1C, iii) rotational mode (R)–microrobot rotates around an external origin as in Figure 1D, and iv) translational mode (T)–microrobot moves on a linear trajectory as in Figure 1E (see Movie S1, Supporting Information).

**Figure 2:**
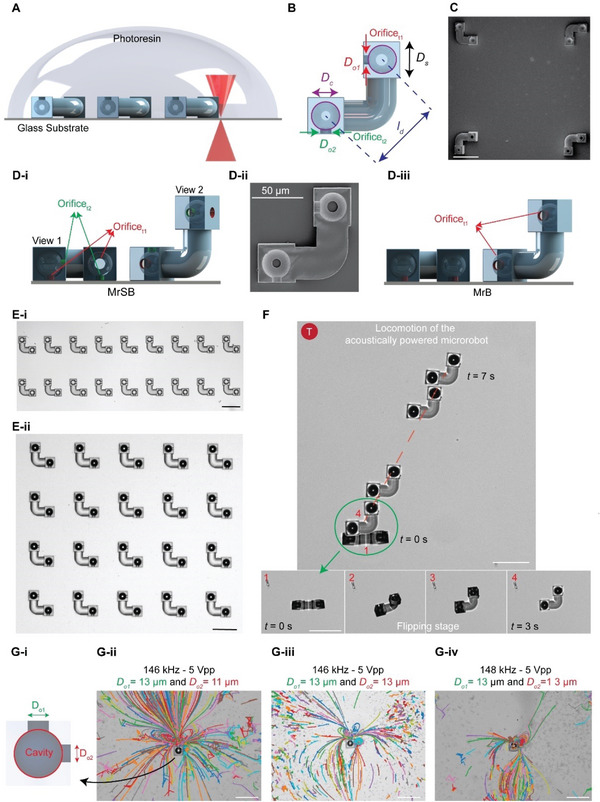
Fabrication and propulsion of the acoustically‐powered microrobot. A) 3D printing of the microrobots using the two‐photon lithography technique. B) Schematics of the microrobot, where *D*_s_ is the base length, *D*_oi_ (*i* = 1 and 2) shows the orifice diameters, and *D*_c_ is the cavity diameter. C) Scanning electron microscopy image of the printed microrobots. Schematics of the D‐i) MrSB and D‐iii) MrB with four and two orifices, respectively. D‐ii) Scanning electron microscopy image of half‐printed MrSB. An array of microrobots E‐i) without and E‐ii) with trapped microbubbles after immersing in a fluidic medium. F) Overlaid experimental images of the robot's propulsion, where the microrobot flips toward the substrate (steps 1 to 4) and starts sliding under the action of the sound wave. G) The microstreaming pattern of three bubbles with 13 and 11 µm orifices excited with a sound wave at *f* = 146 and 148 kHz. Scale bars: 100 µm, unless otherwise stated.

The microrobots are fabricated using a two‐photon polymerization technique, as shown in **Figure** **2**. The microrobots are L‐shaped structures with two 30 µm diameter cavities at the center of cubic bases with 40 µm side lengths. Different‐sized orifices are placed at the center of the void facing the bottom or side of the cubes. Two cubic bases are connected with a curved cylindrical shaft, as shown in Figure 2B (the scanning electron microscopy images are shown in Figure 2C). Microrobots shown in Figure 2D‐i possess two pairs of orifices with 13 and 11 µm diameter. This group of microrobots with a pair of side and bottom nozzles for each cavity inside the L‐shape body is denoted as MrSB (Microrobot with Side and Bottom nozzles). The characterization with scanning electron microscopy was performed to test the quality of the 3D‐printed microrobots, as shown in Figure 2D‐ii. Microrobot shown in Figure 2D‐iii possesses only a pair of 13 µm nozzles at the bottom of every cavity, therefore denoted as MrB (Microrobot with only Bottom nozzles). The third type is similar to the second one; however, a pair of nozzles are positioned at the side of the cavity and thus referred to as MrS (Microrobot with only Side nozzles–Figure S1, Supporting Information).

Due to the surface tension, the microbubbles upon immersion in the liquid medium were immediately trapped inside the spherical cavities (Figure 2E). The formation of bubbles is heavily influenced by the wetting behavior of the polymeric shell when it comes into contact with liquid. To trap air bubbles, the rigid shell is designed with small orifices that prevent the liquid (e.g., water or PBS) from spreading too much and maintain a clear boundary between the air and liquid. This is due to a difference in air pressure and surface tension; liquid molecules have a natural tendency to stick together due to cohesive forces, creating a kind of boundary. This surface tension acts like a barrier, making it difficult for air molecules to escape. Also, the pressure difference inside and outside of the polymeric shell creates a force promoting entrapment and the formation of air bubbles within the cavity upon immediate contact with liquid.^[^ ^39^, ^40^, ^41^, ^42^ ^]^ Figure S3 (Supporting Information) displays the contact angle measurements of the printed resin with a droplet of PBS. Under ultrasound actuation, the microrobots initially flipped toward the substrate; and then performed the desired locomotion mode based on the actuation frequency and amplitude. Figure 2F depicts the actual microrobot's flipping motion and translation mode.

Multimodal locomotion arises due to the diversity of the flow around the microrobot. Tuning the acoustic wave input changes the dominant orifice responsible for the flow profile, i.e., the acoustic streaming of every orifice is pressure‐ and frequency‐dependent. The frequency, amplitude, boundary conditions (e.g., distance from the rigid wall), and the ratio of orifice size to the bubble diameter (*D*_oi_ */D*_c_) are the four main parameters that affect the orifice microstreams. While orifice size and boundary condition are used for tunable actuation, sound wave control inputs are used to change the bulk acoustic streaming. Interestingly, a combination of these different parameters in our proposed microrobot results in multimodal locomotion with multi‐DOF. Figure 2G shows an example of the effect of the orifice size and wave frequency on the flow pattern around a cavity with two orifices. It is imperative to note that reducing a single orifice dimension from 13 to 11 µm can have a significant impact on the generated flow pattern as shown in Figure 2G‐ii,iii. Also, even the slightest variations in sound wave frequency can result in noticeable changes in the flow pattern, as in Figure 2G‐iii,iv. These findings underscore the critical importance of accounting for all variables when designing systems that rely on precise fluid flow and will be investigated further in upcoming sections.

## 3 Locomotion Characterization

By sweeping the acoustic wave frequency and amplitude, we have observed different locomotion modes. Despite MrSB and MrB, the third microrobot (microrobot with only two side nozzles –MrS) shows mainly no locomotion under the action of the acoustic field. We believe that this could be related to the lack of microstreams beneath the robot (i.e., no nozzles at the bottom of the cavity facing the substrate) and significant adhesion and friction forces with the substrate. The inactivity of MrS indicates the importance of the bottom orifices that should be considered when designing such acoustic microrobots. Apart from the apparent effect of the streaming of the bottom orifices on the bulk flow around the microrobot, which engages to generate diverse flows, the streaming of the bottom orifices reduces the above‐mentioned forces with the substrate. Hereafter, we only focus on MrSB and MrB, which show motility under the excitation of sound waves, to characterize the behavior of the acoustically‐powered microrobots under different pressure distributions. Additionally, we considered four main parameters under the different acoustic wave inputs to characterize the acoustically‐powered microrobot performance, listed as: the translational speed (*V_T_*), average speed (*V*_A_), radius of curvature (*RoC*), and distance traveled (*d/D*). The distance traveled shows the ratio of the shortest linear path to the total travel path (see “Locomotion characterization” in Experimental Section).

### 3.1 Multimodal Locomotion Performance of MrSB

**Figure 3:**
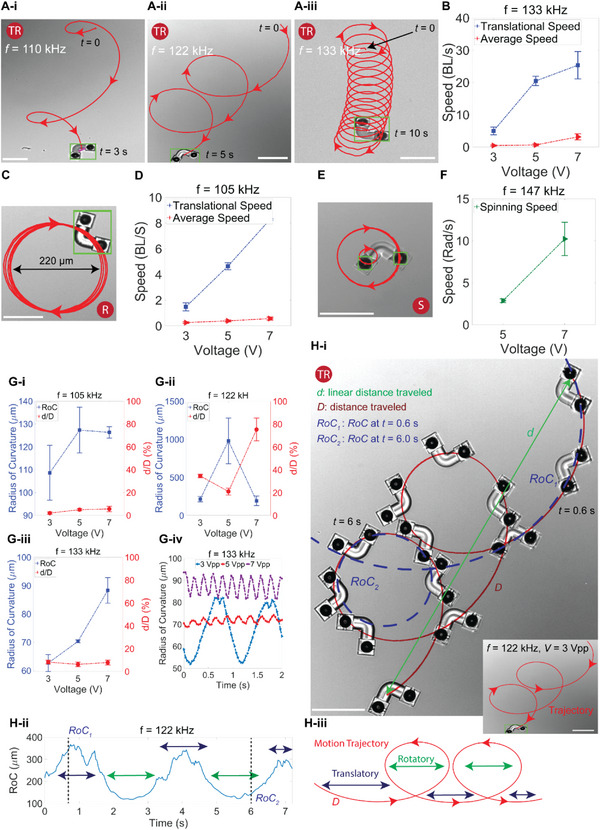
Locomotion characterization of the microrobots with a pair of side and bottom nozzles (MrSB). A) Motion tuning using the acoustic wave frequency, where the microrobot switches its motion from a translational‐ to rotational‐dominated trajectory in the TR mode at A‐i) *f* = 110 kHz, A‐ii) *f* = 122 kHz, and A‐iii) *f* = 133 kHz. B) The average linear and translational speed of the microrobots under different intensities of sound waves, reaching up to 30 BL s^−1^. C) Rotation mode of the microrobot at sound wave frequency of *f* = 105 kHz and *V* = 3 Vpp. D) The average linear and translational speed of the microrobots at *f* = 105 kHz. E) The spinning trajectory of the microrobot at *f* = 147 kHz. F) Spinning velocity of the microrobots at different voltage amplitudes with a sound wave frequency of *f* = 147 kHz. G) The effect of wave frequency and amplitude on the radius of curvature (*RoC*) and *d/D* at G‐i) *f* = 105 kHz, G‐ii) *f* = 122 kHz, and G‐iii) *f* = 133 kHz. G‐iv) Radius of curvature over time for a TR mode at different voltages and *f* = 133 kHz. H) *RoC*, *d*, and *D* representations for a microrobot moving on a spiral trajectory. Scale bars: 100 µm. The error bars show the standard deviation of three different tests.

We observed a broad spectrum of movement trajectories for MrSB at the bubble resonant and non‐resonant actuation frequencies (see “Resonance and actuation frequency” in Experimental Section). Different bulk streaming patterns of four orifices, varying in size and arrangements for two bubbles, make it possible to tune the rotation and translation characteristics of a microrobot's locomotion (**Figure** **3**). As the excitation frequency increased from 110 kHz to 133 kHz, the radius of the rotational portion of the trajectory changes from 60 to 125 µm and then becomes 70 µm (Figure 3A). The translational and rotational (TR) locomotion mode is the most occurring for MrSB type as it appears in a wide range of frequencies and power amplitudes; however, changing the frequency from 133 to 105 or 147 kHz would switch the TR mode to the R (Figure 3C,D) and the S mode (Figure 3E,F), respectively. Considering the microrobot's length scale (*l*_d_), excitation frequency range (70 to 250 kHz), and acoustic wavelength, the attenuation length (∝*1/f^2^*) is large enough to neglect the Eckart radiational forces and only consider the streaming forces^[^ ^43^ ^]^ (see section “Locomotion mechanism of the acoustically powered microrobots” in Experimental Section). Hence, by sweeping the acoustic wave frequency, we found the microrobot's highest speed at TR mode to be ≈30 body lengths per second (BL s^−1^) at *f* = 133 kHz, which corresponds to 3000 µm s^−1^ at a voltage input of 7 Vpp for the piezoelectric transducer. Increasing the power amplitude of the transducer increases the oscillation amplitudes of the sound waves and improves the power transfer to the microrobot at longer distances. However, due to the complexity of flow and their effect on microrobot dynamics, increased power does not necessarily and proportionally increase the microrobots' locomotion speed (Figure S4, Supporting Information). This can be attributed to the streaming interface of different nozzles and two oscillating bubbles.

Like the TR mode, the spinning (S) mode is achievable in different acoustic waves, and the spinning velocity ranges between 90 rpm at *f* = 147 kHz and *V* = 7 Vpp up to 600 rpm at *f* = 275 kHz and *V* = 5 Vpp (Movies S1 and S2, Supporting Information). Additionally, the power dependency of multimodal locomotion is noticeable at different frequencies. Changing the acoustic intensity directly affects the *RoC* and *d/D*, e.g., at *f* = 105 kHz, locomotion is switched from pure R (*V* = 3 Vpp) to the TR (*V* = 5 and 7 Vpp) mode, indicated by the stepped *d/D* = 0% at *V* = 3 Vpp to *d/D* ≈ 8% at *V* = 7 Vpp (Figure 3G‐i). At the sound wave frequency of *f* = 122 kHz, the microrobots switch locomotion from TR to spin and translate (ST) mode by increasing the power to *V* = 7 Vpp (Figure 3G‐ii). This ST mode is indicated by very low *RoC* (due to spinning) and high *d/D*, which shows the dominancy of the translation motion (trajectories of the microrobots at wide ranges of frequencies are shown in Figure S5, Supporting Information). While, the *RoC* is more uniform for both R and S modes over time, switching the mode to TR increases the deviation of the *RoC* over a single period (Figure S5, Supporting Information). Despite other frequencies, in which the robot switches the locomotion mode, the microrobot exhibits only TR mode at *f* = 133 kHz, observed with a uniform *d/D* at different voltages (Figure 3G‐iii). Additionally, larger *RoC* oscillation at *V* = 3 Vpp in Figure 3G‐iv is attributed to the experimental measurement limitations of the *RoC*. We use five data points to calculate the radius of curvature at every time step; slower robots possess smaller increments over time that result in higher standard deviations. Figure 3H shows the instantaneous radius of curvature of the microrobots excited with the acoustic wave at *f* = 122 kHz and *V* = 3 Vpp. Higher *RoC* values in one period (Figure 3H‐ii) is due to longer translation portion of the trajectory shown by dark‐blue arrows (examples of tunable locomotion behavior of the microrobot are presented in Movie S3, Supporting Information).

### 3.2 Translation and Rotatory Modes with Only Bottom Cavities—MrB

**Figure 4:**
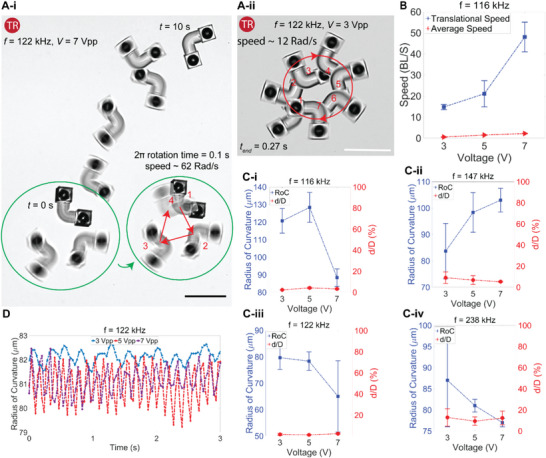
Locomotion characterization of the microrobots with only bottom nozzles for each cavity inside the L‐shape body (MrB). A) Dominant TR mode of the MrB; changing the wave intensity changes the rotational speed, and not the locomotion mode. B) The translational and average speed of the microrobots under different voltages, reaching up to 50 BL s^−1^. C) The effect of wave frequency and amplitude on microrobots’ radius of curvature and *d/D*. D) The radius of curvature of the trajectory under different power amplitudes. Scale bars: 100 µm. The error bars show the standard deviation of three different tests.

Similar analyses are conducted for MrB, shown in **Figure** **4**. While MrSB possesses tunable locomotion modes at different sound wave frequency and amplitude, MrB only exhibits the TR mode (Movie S4, Supporting Information). This observation can be attributed to the effect of two extra orifices added to MrSB, which introduces additional DOF. MrB only has two similar‐sized orifices facing the substrate after the flipping stage. The asymmetric geometry of the L‐shaped structure results in asymmetric bulk streams around the microrobot and T and R modes. This also explains the dominance of the TR mode for the MrSB. However, the lack of the additional side orifices for MrB, limits the diversity of the microstreaming patterns, and consequently, restricts the locomotion mode to only TR mode. Furthermore, we have demonstrated that the influence of sound wave frequency and amplitude on MrB is solely noticeable at its speed (Figure S6, Supporting Information). The *RoC* and *d/D* for MrB are almost constant over different actuation wave inputs (Figure 4C). Additionally, comparing the velocities of two proposed mobile microrobots (MrSB and MrB) shows that adding an extra orifice to the cavity decreases the microrobots' speed. Speeds of MrB can reach up to 55 BL s^−1^ (Figure 4B), which corresponds to 4500 µm s^−1^, and a rotational speed of 10 Hz (Figure 4A‐i), which are higher compared to the MrSB. In short, our results indicate that the locomotion behavior of the MrB is changeless to the increased power (Figure 4D). Additionally, the only contribution of the power is observed for the locomotion speed, e.g., in Figure 4A, the rotational velocity increased from 3 to 10 Hz, while increasing the power from *V* = 3 to *V* = 7 Vpp at *f* = 122 kHz, with a uniform locomotion mode.

## 4 Flow Characterization of the Acoustic Streaming

**Figure 5:**
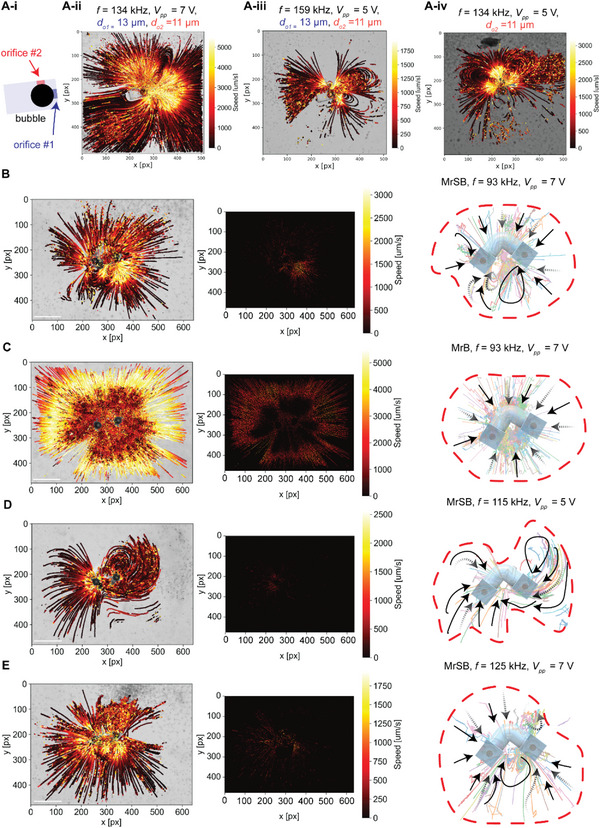
The microstreaming of the acoustically‐powered microrobot. A) Acoustic streaming of a bubble trapped in a cavity with two and one orifices at sound wave frequencies of *f* = 134 and 159 kHz. Particle trajectory and its instantaneous speed (left), mean spatial speed of particles over time (middle), and schematics of the bulk flow around the microrobots (right) for B) MrSB and C) MrB at the sound wave frequency of *f* = 93 kHz. D,E) Particle trajectory and mean speed with curved flow patterns for MrSB at *f* = 115 and 125 kHz. Particle trajectories in (B–E) correspond to ≈25 to 30 µm *z* planes above the substrate. Faded dashed lines represent the trajectory lines with a *z*‐elevation. Scale bar: 100 µm.

The vibration of the bubble‐air interface under the sound wave results in acoustic streaming. The maximum particle velocity and the bulk flow trajectory were analyzed with a custom‐made particle tracking algorithm for the microrobots anchored to the substrate. **Figure** **5** shows the instantaneous speeds and trajectories of 2 µm diameter polystyrene particles for a single bubble with one and two orifices and for the microrobots with two coupled bubbles. The heat maps in Figure 5 show the spatial mean for Δ*t* = 5 s. Our results (Figure 5A; Figures S12–S21, Supporting Information) show having two orifices on a cavity alters the uniform counter‐rotating vortex flow of a bubble with the single orifice to a combination of vortex and jet flow with symmetric and asymmetric patterns. However, the interaction between two oscillating bubbles on the same microrobot further expands the diversity of the bulk flows; curved flow patterns emerge when two similar‐sized bubbles with different combinations of nozzles interact (Figure 5B). For MrB, with only bottom nozzles, the single bulk flow was observed at different sound wave frequencies and amplitudes, where the flow was sucked toward the center and pumped upward as shown in Figure 5C and Figure S12 (Supporting Information). This is in line with the observations for a single bubble with a single orifice. However, for MrSB with four nozzles, bulk patterns are tunable based on the sound wave frequency and amplitude (Figure 5B,D,E; Figure S13, Supporting Information). These different patterns change the thrust force acting on the microrobot and initiate a unique locomotion mode. Changing the sound wave frequency and amplitude not only changes the flow pattern it also directly alters the spatial instantaneous stream speed around the microstructure; consequently, changing the momentum and distributed acting forces on the microrobot (acoustic jet streaming of the microrobots are presented in the Movie S5, Supporting Information). Figure S14 (Supporting Information) displays simulated 3D patterns of two oscillating trapped bubbles in microcavities and four nozzles (MrSB), showcasing a 3D curved streaming pattern.

The size of the orifice directly affects its actuation frequency and the flow velocities. As a rule of thumb, decreasing the size of the orifice, while keeping the bubble diameter fixed, shifts the actuating frequencies of the orifice to higher frequencies. Additionally, the jet‐like flow of the oscillating bubble reforms to a symmetrical vortex at the nozzle as the sound wave frequency reaches to bubble's natural frequency for an orifice parallel to the substrate (Figure S15, Supporting Information). On the contrary, the orifice facing the substrate turns the flow pattern to a more random 3D flow with no particular pattern (Figure S16, Supporting Information). The combination of two orifices on a single cavity (one facing the substrate and one parallel to it) results in the combination of jet‐like with vortices, and random 3D flows at broad ranges of frequencies (Figure S17, Supporting Information). The combination of two different‐sized orifices makes it feasible to generate additional types of bulk flows around the microbubble, where the contribution of every orifice on the bulk stream is controllable using sound wave input as in Figure 5A and Figure S18 (Supporting Information).

Additionally, our analyses indicate that increasing the size of the orifice enhances the jet‐like pattern while decreasing the maximum streaming speed, e.g., actuating a bubble with a single orifice with diameters of 11 and 13 µm at *f* = 134 kHz, *V* = 5 Vpp will result in maximum stream velocity of 3000 µm s^−1^ for orifice size of 11 µm (Figure S20, Supporting Information) and 2000 µm for the orifice size of 13 µm (Figure S15, Supporting Information). Furthermore, our results indicate that at a fixed sound wave frequency, *f* = 134 kHz, increasing the power enhances the pattern formation while keeping the maximum stream velocity constant (Figures S19 and S20, Supporting Information).

A combination of two different‐sized orifices on a cavity with different boundary arrangements makes it possible to alter the flow from 3D random patterns at low power and frequencies to a mix of jet‐like and vortex flows at higher sound wave frequency and intensity. In summary: i) the orifice facing the substrate generates 3D random flows with no uniform patterns, ii) the orifice parallel to the substrate results in streams with jet‐like flow, vortex flow, or patterns with a combination of them; iii) having two orifices on a cavity, one facing the substrate and one parallel to it results in a mixed flow of 3D random, and vortex flow with enhances jets; iv) increasing the orifice‐size for a cavity enhances the jet‐like flows; v) increasing the power input to the piezoelectric transducer generates tangible flow patterns with a minimal effect on maximum stream velocity for a cavity with two orifices (flow analyses of single bubble trapped in a cavity with different nozzle sizes, orientations, and varying sound waves are shown in Figures S15–S21, Supporting Information).

## 5 Bioanalyses and Biomedical Applications

### 5.1 Cell‐Lysing

**Figure 6:**
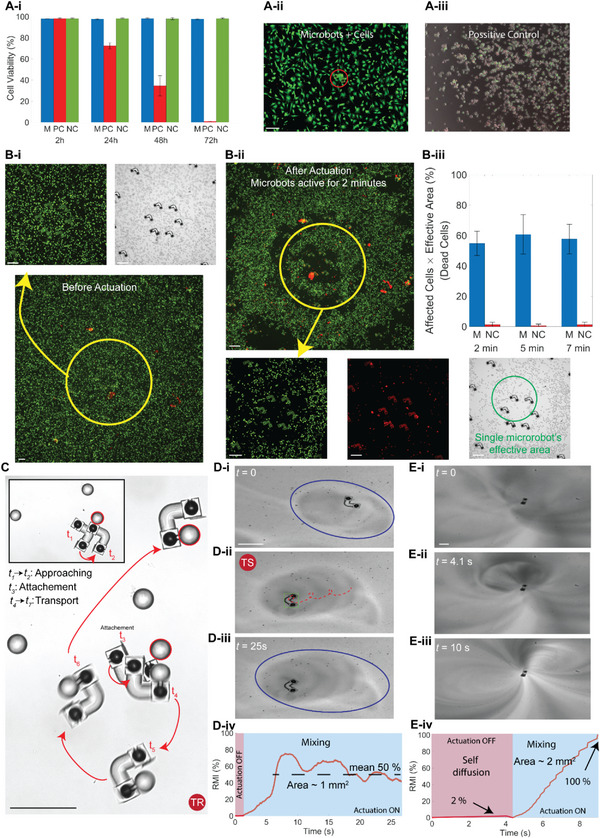
Toxicity analyses, cell‐lysing, particle manipulation, and mixing performance of the acoustically‐powered microrobots. A‐i) Biocompatibility analyses of the microrobots with sensitive HMSC cells. M showing the medium, including cells and microrobots, PC indicating the positive control tests with 20% DMSO, and NC indicating the negative control tests which involve medium filled with cells and no additional additives. A‐ii,iii) Microscope images of the M and PC tests after 72 h. B) Cell‐lysing tests of the microrobots with high flow rates and actuating periods of 2, 5, and 7 min. B‐i,ii) Green, red, and bright light images of the setup before and after 2 min actuation. B‐iii) Cell‐lysing results showing the affected human breast cancer cell (dead cells) after the actuation period. M indicating a chamber filled with microrobots and cells; NC shows sets of experiments at similar actuating frequency and amplitude for a chamber filled with only cells and without microrobots. C) Particle manipulation; attachment and transport periods for a robot moving at TR mode. D) Mixing two fluids with a microrobot operating at TR mode. 50% mixing is achievable within the image field of view in 25 s. E) Mixing fluid at a high flow rate with an anchored microrobot, where mixing reaches 100% in 6 s compared to self‐diffusion which reaches up to 2% in 5 s. Scale bars: 100 µm. The error bars show the standard deviation of three different tests.

Microsystems powered by sound waves have high flow control resolution that can potentially tackle the challenges faced by conventional microrobots in numerous applications, including drug delivery, on‐command drug diffusion, and mixing. We show a set of biomedically relevant applications here. Biotoxicity analyses were conducted to analyze the toxicity of microstructures manufactured with 2PP photoresin IP‐S. The biotoxicity of the printed structures was investigated using human Mesenchymal Stem Cells (hMSC), which are reported to be more sensitive to any toxicity in the medium compared to other organisms, such as Human Umbilical Vein Endothelial Cells (HUVEC). Cell viability of three different groups was analyzed; negative control (i.e., cells with only medium and without any additives), positive control (i.e., cells with 20% DMSO), and acute toxicity (i.e., medium with cells and non‐active microrobots). Analyses were conducted for up to 72 h inside the incubator, and the results are presented in **Figure** **6**. The analyses indicate that printed non‐active microrobots show no damage to the cells. Figure 6A‐ii,iii shows the acute toxicity and positive control tests after 72 h.

Next, the interaction of the active microrobots with cancerous cells is tested. Figure 6B shows the cell‐lysing of HUVEC in brief periods, i.e., 2, 5, and 7 min. Figure 6B‐i,ii shows the microscopic green (live), red (dead), and bright channel images of the microrobots with cells before and after actuation. Lysing tests are done for 2, 5, and 7 min, then the number of dead cells is counted. It is important to note that, due to a significant difference between the chamber volume and the microrobots’ covered area, the live/dead analyses are corrected using effective area parameters (see “Cell culture and bioanalyses” in Experimental Section). The cancerous cell‐lysing results show a minimal difference between different actuation durations; on average, 55% lysing is achievable using the acoustically actuated microrobots without any additives to the medium or heating. Negative controls for these tests are similar experiments, where the piezoelectric disk is actuated at a similar frequency and amplitude; however, the microrobots are emptied from the chamber filled with PBS and cells. Figures S22 and S23 (Supporting Information) display setup, before and after microscope images, and the arrangement of microrobots for lysing tests. Additionally, morphological alterations were observable during the lysing process, as shown in Movie S6 (Supporting Information).

### 5.2 Particle Manipulation

Considerable acoustic radiation and streaming forces (Bjerknes forces) of an oscillatory bubble interact with the surrounding particles and could be used to manipulate small objects.^[^ ^29^ ^]^ Radiation force and the oscillatory bubble and particle interactions are size‐dependent. While smaller particles with low density follow the streamlines generated by the bubble oscillation, larger particles become trapped in the resonating bubble.^[^ ^44^ ^]^ Taking advantage of the same phenomena, we demonstrated the trapping, collection, and transport of 30 µm polystyrene particles embedded in the fluid medium shown in Figure 6C and Movie S7 (Supporting Information).

### 5.3 Mixing

Microbubbles are an indivisible part of microrobots with oscillatory‐bubble propulsion, capable of generating very high flow rates that could be used for active macromixing and on‐demand diffusion. Selectable actuation of different‐size orifices and bubbles in a single microstructure makes it possible to separate propulsion, mixing, and diffusion modalities.

The mixing efficiency is a crucial parameter for microfluidic devices. A standard method to measure the mixing efficiency is based on the intensity of segregation, which has been used in this study to evaluate the mixing performance of the microrobots (see “Mixing characterization” in Experimental Section). The results in Figure 6D,E show the increased mixing efficiency up to 50% and 100% within a concise period for a mobile and anchored microrobot to the substrate, respectively. The mixing efficiency increases from 2% self‐diffusion to 100% as the tapped bubble inside the cavity on a pole oscillates, where the interest area is ≈2 mm^2^. The microrobot moving inside the medium was capable of reaching a mixing ratio of 50% on average within 20 s for an observed area of ≈1 mm^2^. The mixing performance of the microrobots is presented in Movie S8 (Supporting Information).

## 6 Discussion and Conclusion

Acoustically‐driven microrobots are safe and reliable tools for biomedical applications. In most cases, the acoustically‐powered microdevices do not require specific conditions, such as high pressure, high temperature, or particular material type for propulsion or manipulating the environment.^[^ ^45^, ^46^ ^]^ On top of that, diversity in the range of flow patterns and velocities of an oscillatory bubble, excited with a traveling sound wave, has led to vast fields of applications in bioengineering^[^ ^47^, ^48^ ^]^ and life sciences.^[^ ^49^ ^]^ Despite most untethered synthetic microrobots that follow the scallop theorem^[^ ^50^ ^]^ to create nonreciprocal motion at low Reynolds numbers,^[^ ^51^, ^52^, ^53^ ^]^ bubble‐based propellers are capable of operating at vast ranges of flow speeds and low to medium Reynolds number (0.01<*Re*<300).^[^ ^29^, ^30^, ^54^ ^]^ In this study, we demonstrated a propulsion mechanism that takes advantage of different bulk flows of two coupled microbubbles to perform multimodal locomotion. The heterogeneous oscillation of two nozzles of different diameters in a single cavity and the interaction of two adjacent bubbles in a single microstructure grant the possibility of generating different flow patterns and instantaneous speeds, thus enabling multi‐modal locomotion.

The directional flow of an oscillating bubble establishes the basis of the bubble propulsion mechanism. Current acoustically‐powered microrobots are limited to a single mode of locomotion because of the inflexibility of the acoustic micro‐streaming patterns.^[^ ^29^, ^32^ ^]^ It is important to note the significance of the bulk streaming of two coupled oscillating bubbles trapped in a cavity with different‐sized orifices in the multimodal locomotion presented in this work. Acoustic streaming of an oscillatory bubble is both amplitude and frequency‐dependent. Also, one evident outcome of increased sound wave intensity in such single DOF microrobots with a single orifice is the observable higher oscillation amplitudes corresponding to higher streaming speed, consequently, faster microrobots. Our approach enables a high degree of controllability over the flow patterns by controlling both the intensity and frequency of the sound wave leading to multimodal locomotion.

The multi‐input characteristic of the microrobot presented in this work adds multiple DOF to the acoustically powered microdevices that were not achieved before. Aside from sound wave intensity and frequency, acoustic streaming of an oscillatory bubble highly depends on the surrounding boundaries, and their distances (*l*_db_). It has been theoretically shown that the presence of a rigid wall changes the amplitude and the phase of the bubble oscillations, enhancing the acoustic microstreaming.^[^ ^55^ ^]^ Later, Bertin et al.^[^ ^34^ ^]^ conducted experiments with a microbubble inside a spherical capsule attached to a pole and showed that the recirculating streamlines appear around the capsule near the wall boundary and the microstreaming of an oscillatory bubble dramatically changes when the height of the pole is increased from 10 to 30 µm. The microcavities in our study possess two orifices, one facing the substrate, and the other parallel to the substrate. Regardless of the sound wave and the size of the orifices, two orifices with different boundary conditions (e.g., *l*_db_) will generate distinct acoustic micro‐streams. Additionally, in our proposed microrobot, two orifices on the same bubble have different *l*_db_ values, and differ in size and arrangement, which leads to diverse 3D flow patterns at different sound waves. Two general criteria concerning the acoustic streaming of the presented microrobots are as follows: i) for a microrobot with two nozzles on each cavity (MrSB), the flow between two bubbles is mainly a vortex linking two bubbles, and the rest of the bulk stream is a combination of the jet‐like and rotating vortex; ii) for a microrobot with only single bottom nozzles on each cavity (MrB), the flow is always directed to the center of two bubbles and then pumped vertically to higher z amplitudes. Additionally, in the context of our study, we've observed a nuanced relationship between sound wave frequency and emergent locomotion modes. Contrary to expectations, we've found that changes in sound wave frequency do not consistently correlate with specific locomotion modes. This lack of clear correlation suggests that the appearance of various locomotion modes might seem random. We attribute this unpredictability to the intricate and non‐patterned nature of fluid flows around the robot relative to frequency variations. These findings highlight the complex interplay between sound waves, fluid dynamics, and locomotion, prompting further investigation into the intricate mechanisms underlying these behaviors.

Besides the multimodal locomotion capabilities of the acoustically‐powered microrobots, we showed that the high degree of flow control enables different biomedical functions with the proposed microrobot design; i.e., cancerous cell‐lysing without the requirements for additional additives, microparticle manipulating using radiation force and streaming forces, and fluid mixing. Cell lysis or cellular disruption is a method in which the outer cell boundary is broken down or destroyed to release inter‐cellular materials or kill them.^[^ ^56^ ^]^ Multiple strategies have been established to lyse cells on a macro or micro scale that can be grouped into two main categories: mechanical or non‐mechanical.^[^ ^57^ ^]^ Non‐mechanical methods require chemical (e.g., alkali chemicals or detergents), biological additives (enzymes), or use physical shocks, such as heating, osmotic, or cavitation to lyse cells. On the other hand, relatively more popular mechanical methods are based on high shear forces on the membrane to perform a similar task. Mechanical methods are very efficient in lysing a wide range of cells; however, problems, such as heating of sample volume, degradation of cellular products, cell debris, and higher costs, limit their application. Microrobots in this study are efficient and inexpensive methods capable of generating high flows (*V* ≥ 7 Vpp), which can be used to perform similar tasks and address some of the problems mentioned above. Our tests indicate that there is minimal difference between the lysing periods and on average lysing was achieved by up to 50%.

The micromixers significantly impact microfluidic devices that target various applications, such as biomedical diagnostics, drug mixing, and food safety control. Unlike macroscale mixing, which often relies on convection, mixing on a micro‐scale is achieved with external turbulences or microstructures to increase the mass transfer efficiency. However, low flow rates on a small scale, consequently a low Reynolds number (*Re* ≪ 1), indicate the dominance of slow self‐diffusion and low efficiency.^[^ ^58^ ^]^ Additionally, many microrobots have simple structures that rely on passive self‐diffusion of the drug and low efficiency with a small diffusion region and an additional external stimulus to diffuse the drug. A vast range of flow rates (0.01<*Re*<300) with different stream patterns of the bubbles under the excitation of the sound wave shapes the mixing property of the proposed microrobots and enables active mixing of different fluids with distinct properties locally and in a larger macro environment.

The design of the microrobot could be modified to limit the flow of an extra third nozzle on one cavity to achieve on‐command drug release. Also, adjusting the microrobots’ body with different materials, such as gelatin and fibronectin, could be used to encapsulate cargo (e.g., drugs) and deliver to target locations precisely.^[^ ^20^ ^]^ The presence of the bubble within the structure of microrobots enhances their controllability and detection under different imaging modalities, similar to the contrast agents,^[^ ^59^ ^]^ which is crucial for their presence in minimally‐invasive interventions. Alternately, the directional control of untethered artificial microrobots plays a vital role in their future medical applications. Steering of such acoustically powered microrobots is readily achievable by integrating magnetic nanoparticles within the body during or directional‐sputter‐coating after the printing process. Also, the anisotropic magnetic layer facilitates in‐place torque generation to steer acoustically powered microrobots using coil setups; we believe the conjunction of magnetic directional steering and acoustic actuation will exhibit new locomotion modes to explore. Future work will focus on the collective behavior of multi‐bubble microrobots and the effect of bubble‐induced acoustic streams on cells at different ranges of Reynold numbers, and Computaional Fluid Dynamics analyses to gain a better understanding of the modality associated with acoustic‐streaming.

## 7 Experimental Section

### Fabrication of the Microrobots

Microrobots were 3D‐printed using a commercially available two‐photon polymerization system (Photonic Pro‐Professional GT, Nanoscribe GmbH) with a 63X objective and IP‐S resin in the oil‐immersion mode. The fabrication of the microrobots was realized using 90° hatching angle, 40 mW solid laser power, and 10 × 10^3^ solid scan speed. Next, the microrobots were developed in propylene glycol methyl ether acetate (PGMEA) solution for 40 min to remove the resin inside the spherical cavity followed by 10 min rinsing in isopropanol alcohol (IPA). The microrobots were washed with IPA and air‐dried before every experiment. The microrobots were detached from the substrate using 100 µm needle and transferred into the chamber using double‐distilled H_2_O‐coated micropipette tips.

### Locomotion Mechanism of the Acoustically Powered Microrobots

An acoustically excited free bubble (a bubble without any structure encapsulating it) exhibited mainly two oscillation modes: radial and lateral.^[^ ^60^ ^]^ The Laplace pressure was the primary driving mechanism of the bubble‐based actuation and was balanced with the surface tension at the air–liquid boundary.^[^ ^61^ ^]^ By assuming comparable radial and lateral oscillation amplitudes, radial oscillations were shown to substantially enhance the streaming velocity.^[^ ^62^ ^]^ In the case of a bubble inside a cavity, the spherical cavity reinforced with a polymeric shell limits the air–liquid interface to only the nozzle opening and exposes the air–liquid boundary to normal oscillatory stress. The rest of the air bubble inside the cavity was in contact with the rigid polymeric shell and was at an equilibrium pressure experiencing no normal stress. However, combining two nozzles on a cavity couples two oscillation modes of the bubble, resulting in a combination of vortex flow and significant jets‐like flow around the nozzle under the action of different‐frequency sound waves.

The oscillation amplitudes of two different‐sized orifices on a single cavity were different and are pressure‐ and frequency‐dependent. Different oscillation amplitudes and stream patterns of every nozzle at different sound waves make it possible to induce various forces on the microrobots body, hence, observing multimodal locomotion with multi‐DOF. The following equation could describe the movement of a microstructure under the action of the acoustic field,

(1) $$\ddot{X}=F_{g}+F_{rad}+F_{dr}+F_{st}+F_{b}+F_{f}$$

where ***X*** is the movement vector normalized by mass [m kg^−1^], ***F***_**g**_ is the constant gravitational force, ***F***_**dr**_ is the fluid drag force, ***F***_**b**_ is the buoyant force, and ***F***_**f**_ is the friction and adhesion forces acting on the structure. ***F***_**rad**_ and ***F***_**st**_ are the primary acoustic radiation force acting on the rigid shell due to the traveling wave, and bulk streaming forces, respectively. The force caused by the radiation of the scattered acoustic waves from the rigid polymeric surface (***F***_**rad**_) and the steady streaming forces surrounding the object (***F***_**st**_) propel the microrobot under the acoustic actuation by overcoming the remaining force components of Equation (1).^[^ ^63^ ^]^ The ratio of the acoustic streaming force to the acoustic radiation force mainly lies in the range of ***F***_**st**_ */**F***_**r**_ ≈ 5–45, indicating the dominance of the streaming force over the radiation force, for low amplitude sound waves at the microscale (see Appendix, Section S3, Supporting Information “Radiation and streaming force” for details).

### Resonance and Actuation Frequency

The streaming forces played the dominant role in the multimodal locomotion behavior of the microrobot. The accumulated streaming forces of the nozzles that differ in size or placement determine the overall micro‐streaming patterns and the locomotion mode. The acoustic streaming forces were most efficient when the bubble was excited at its resonance frequency. The resonance frequency of the bubbles in the design with a small oscillation amplitude could be estimated using the Rayleigh–Plasset^[^ ^61^ ^]^ equation which was in the range of 220 kHz; and most of the experiments were done within the frequency range that the piezoelectric transducer has high power output (see Appendix, Section S1, Supporting Information “Resonance frequencies and pressure map”). Furthermore, bubble stability tests were performed within a similar frequency range (Movie S9, Supporting Information) and Appendix, Section S2, Supporting Information “Bubble stability” discusses the active and passive stability test analyses.

### Locomotion Characterization

Translational speed (*V*_T_) was defined as the mean of the instantaneous speed of the microrobot. Average speed (*V_A_*) determines the ratio of the shortest distance traveled in a single period over time (*d/t*). The radius of curvature (*RoC*) measures the reciprocal of the instantaneous curvature. Distance traveled (*d/D*), showed the ratio of the shortest linear path from *t* = 0 s to *t* = *t*_end_ (*d*) to the total length of the traveled path (*D*). Intuitively, the linear trajectory was indicated by high *RoC* and *d/D*. The instantaneous speed of the microrobots was calculated by dividing the distance traveled by the time and then normalizing it using *l_d_*, which showed the body length traveled per time unit (BL s^−1^).

### Imaging and Tracking of the Microrobots

The microrobot's initial characterization (e.g., bubble formation, cavity print, and structure shape) and following tests were done using an inverted optical microscope (Nikon Instruments). The microrobot images under a Nikon microscope were captured by a Hamamatsu Orca Flash4 camera (Hamamatsu Photonics) with 4X, 10X and 20X objectives. For flow characterizations, the images were taken using a high‐speed camera (M310; Phantom, Inc.) with 1000 frames per second and 200 ms exposure time. The images were then analyzed using a custom‐made Python code. Microrobot tracking was performed using a custom‐made MATLAB script (MATLAB and regionprops Toolbox Release 2020b, The MathWorks, Inc., Natick, Massachusetts, United States.) for feature detection and trajectory linking. Movie S10 (Supporting Information) showed the detection and measurement distribution among several samples.

### Acoustic Setup and Hydrophone Measurements

To characterize the microrobot's locomotion, microchannels made of acoustically transparent polydimethylsiloxane (PDMS) were made and filled with phosphate‐buffered saline (PBS). The acoustically transparent characteristic of PDMS prevents standing waves from forming within the chamber. A piezoelectric transducer (Murata Piezo Buzzer Diaphragm, Surface Mount, External Dia. 12 mm) was attached to the vicinity of the chamber on a glass slider. Under the sinusoidal input to the transducer, the acoustic waves were transferred to the liquid chamber through the glass slide. PDMS microchambers are manufactured using standard soft lithography and bonded on the glass slide after ozone plasma treatment (25+5 min and 80° C). Microrobots locomotion characterization, flow analyses, bubble stability, and mixing tests were done using a ring‐shaped PDMS chamber with an inner diameter of 12 mm. Cell‐lysing experiments were done using a 6 mm in diameter chamber to decrease the chamber volume and increase the number of cells exposed with individual microrobots. A 14 mm PDMS cab was used to enclose the chambers after transferring the microrobots and filling the chamber with PBS. A function generator was used to actuate the piezoelectric transducer with arbitrary sinusoidal waves at different frequencies and amplitudes.

For pressure measurements, a calibrated needle hydrophone of 500 µm tip diameter (NH0500, Precision Acoustics Ltd.) was used. The hydrophone was placed in the origin of the chamber and above the glass substrate and moved using a motorized XYZ stage. The time domain signals from the driving voltage and acoustic pressure were recorded using a mixed domain oscilloscope (MDO4024C, Tektronix Inc.). Then, the collected signals were analyzed, and the peak amplitudes for the driving voltage and the corresponding acoustic pressure amplitude at different frequencies were obtained (Figure S7, Supporting Information). PBS with 2 µm diameter polystyrene beads was used to characterize the fluid flow and measure the flow speed around the microbubbles using a custom‐made particle tracing velocimetry algorithm.

### Mixing Characterization

The intensity standard deviation of the pixel was used as the mixing index (*RMI* = 1 − Ω/Ω_0_) to evaluate the mixing efficiency where

(2) $$\begin{array}{c} \Omega =\sqrt{\frac{1}{N}\sum_{i\;=\;1}^{N}{\left(I_{i}-\;\langle I\rangle \right)}^{2}},\\ {\;\Omega }_{0}=\sqrt{\frac{1}{N}\sum_{i\;=\;1}^{N}{\left(I_{0i}-\;\langle I\rangle \right)}^{2}} \end{array}$$

and *N* is the total number of pixels, *i* analyzed pixel, *I*_i_ grey intensity, *I*_oi_ intensity of pixel *i* at *t* = 0 s, and <*I*> is the average intensity of the region of interest. *RMI* = 0 indicates no mixing, while *RMI* = 100% means perfect mixing over the whole area of interest.^[^ ^64^ ^]^

### Cell Culturing

For biocompatibility analyses, Human Mesenchymal Stem Cells (HMSC, LONZA) were cultured in high‐glucose Dulbecco's Eagle Medium (DMEM, Gibco) supplemented with 20% fetal bovine serum (FBS, Gibco) and 1% penicillin/streptomycin (P/S, Gibco) in standard culture conditions, 37 °C and 5% CO_2_ humidified atmosphere. Cells were passaged and used for experimentation within passages number 2 to 10. The cells were removed with 0.25% Trypsin (Gibco). For cell‐lysing tests, Human Breast Cancer Cell (SK‐BR‐3, American Type Culture Collection) were cultured in high‐glucose Dulbecco's Eagle Medium (DMEM, Gibco) supplemented with 10% fetal bovine serum (FBS, Gibco) and 1% penicillin/streptomycin (P/S, Gibco) in a standard culture condition. Cells were passaged up to passage 12. Cells were removed with 0.25% Trypsin (Gibco).

### Biocompatibility and Cell‐Lysing Tests

A 96‐well clear bottom plate (Corning) was seeded with HMSC cells at different concentrations for different exposure times. The samples were all tested with three replicates. Exposure times were set at 2 h with 10K cells, 24 h with 10K cells, and 48 h with 5K cells. The cells were allowed to incubate at standard culture conditions for 3 h to allow for appropriate attachment. After the attachment period, the microrobots were added to the wells to begin acute toxicity tests. Once each exposure period had occurred, the Live/Dead Cell Staining Kit was prepared by warming it to room temperature. Then after creating a 2X concentration of the staining buffer, the reagent was added to the wells. The 96‐well plate was placed back into the incubator for 30 min, after which the plate was examined microscopically. Images of live and dead cells were obtained using a fluorescent microscope (Nikon Eclipse Ti‐E, Tokyo, Japan).

The SK‐BR‐3 cells were removed from the culture flask by standard sub‐culturing methods at ≈80% confluency. After centrifugation, the live staining kit reagent was added to the cells in suspension for sufficient time for staining. Then to remove any residual factors, which may interfere with bubble formation, the cells were centrifuged and resuspended with dead stain dye in DPBS (Dulbecco's Buffered Saline Solution). The cell concentration was set to 333333 cells per mL of solution. This concentration was chosen to allow accurate analysis of live and dead cells after the lysis treatment by microrobots. The cells were then placed in the actuation chamber for cell lysis experiments. These experiments occurred over different conditions and periods. The conditions were set as without and with actuation, and time exposure was set for 2, 5, and 7 min periods.

The number‐of‐live‐Cells to number‐of‐Dead‐Cells ratio was corrected using (*Area*_chamber_)/(*N*×*Area*_m_) to compensate for the considerable difference in the chamber volume and affected area by microrobots. *N* shows the number of robots inside the chamber. After conducting flow analysis tests, it was found that a single robot was effective within a 0.2 mm^2^ range. This area could be compared to a circle with a diameter of 500 µm that was centered around the microrobot.

## Conflict of Interest

The authors declare no conflict of interest.

## Author Contributions

N.M., A.A., and M.S. proposed and designed the research; N.M. performed the research; N.M. and D.S. analyzed the data; A.A. and G.G. contributed to the data interpretation and reasoning; N.M. wrote the paper; M.S. and R.K. supervised the research; and all authors participated in manuscript editing.

## Supporting information

Supporting Information

Supplemental Movie 1

Supplemental Movie 2

Supplemental Movie 3

Supplemental Movie 4

Supplemental Movie 5

Supplemental Movie 6

Supplemental Movie 7

Supplemental Movie 8

Supplemental Movie 9

Supplemental Movie 10

## Data Availability

The data that support the findings of this study are available in the supplementary material of this article.

## References

[1] M. Garcia‐Paris , S. M. Deban , J. Herpetol. 1995, 29, 149.

[2] R. H. Armour , J. F. V. Vincent , J. Bionic Eng. 2006, 3, 195.

[3] N. F. Zeng , W. D. Ristenpart , Biomicrofluidics 2014, 8, 064123. 25553197 10.1063/1.4904058 PMC4265125

[4] Z. Budrikis , Nat. Rev. Phys. 2020, 2, 461. 10.1038/s42254-020-00250-w PMC7528152 33728405

[5] C. K. Schmidt , M. Medina‐Sánchez , R. J. Edmondson , O. G. Schmidt , Nat. Commun. 2020, 11, 5618. 33154372 10.1038/s41467-020-19322-7 PMC7645678

[6] Z. Huang , G. Chi‐Pong Tsui , Y. Deng , C.‐Y. Tang , Nanotechnol. Rev. 2020, 9, 1118.

[7] X. Z. Chen , B. Jang , D. Ahmed , C. Hu , C. De Marco , M. Hoop , F. Mushtaq , B. J. Nelson , S. Pané , Adv. Mater. 2018, 30, 1705061. 10.1002/adma.201705061 29443430

[8] M. Sitti , D. S. Wiersma , Adv. Mater. 2020, 32, 1906766. 10.1002/adma.201906766 32053227

[9] N. O. Dogan , H. Ceylan , E. Suadiye , D. Sheehan , A. Aydin , I. C. Yasa , A.‐M. Wild , G. Richter , M. Sitti , Small 2022, 18, 2204016. 10.1002/smll.202204016 36202751

[10] X. Lin , Z. Wu , Y. Wu , M. Xuan , Q. He , Adv. Mater. 2016, 28, 1060. 26421653 10.1002/adma.201502583

[11] Y. Wu , T. Si , C. Gao , M. Yang , Q. He , J. Am. Chem. Soc. 2018, 140, 11902. 30176727 10.1021/jacs.8b06646

[12] L. Ricotti , B. Trimmer , A. W. Feinberg , R. Raman , K. K. Parker , R. Bashir , M. Sitti , S. Martel , P. Dario , A. Menciassi , Sci. Rob. 2017, 2, aaq0495. 10.1126/scirobotics.aaq0495 33157905

[13] L. Yang , L. Zhang , Annu. Rev. Control Robot. Auton. Syst. 2021, 4, 509.

[14] A.‐I. Bunea , D. Martella , S. Nocentini , C. Parmeggiani , R. Taboryski , D. S. Wiersma , Adv. Intell. Syst. 2021, 3, 2000256.

[15] F. Soto , E. Karshalev , F. Zhang , B. Esteban Fernandez De Avila , A. Nourhani , J. Wang , Chem. Rev. 2022, 122, 5365. 33522238 10.1021/acs.chemrev.0c00999

[16] Y. Xiao , J. Zhang , B. Fang , X. Zhao , N. Hao , Micromachines 2022, 13, 481. 35334771 10.3390/mi13030481 PMC8949854

[17] U. Bozuyuk , E. Suadiye , A. Aghakhani , N. O. Dogan , J. Lazovic , M. E. Tiryaki , M. Schneider , A. C. Karacakol , S. O. Demir , G. Richter , M. Sitti , Adv. Funct. Mater. 2022, 32, 2109741.

[18] Z. Zhang , A. Sukhov , J. Harting , P. Malgaretti , D. Ahmed , Nat. Commun. 2022, 13, 7347. 36446799 10.1038/s41467-022-35078-8 PMC9708833

[19] X. Wang , C. Hu , L. Schurz , C. De Marco , X. Chen , S. Pané , B. J. Nelson , ACS Nano 2018, 12, 6210. 29799724 10.1021/acsnano.8b02907

[20] H. Ceylan , I. C. Yasa , M. Sitti , Adv. Mater. 2017, 29, 1605072. 10.1002/adma.201605072 28004861

[21] M. Sitti , Mobile Microrobotics, MIT Press, Cambridge, MA 2017.

[22] W. Hu , G. Z. Lum , M. Mastrangeli , M. Sitti , Nature 2018, 554, 81. 29364873 10.1038/nature25443

[23] S. Agastin , U.‐B. T. Giang , Y. Geng , L. A. Delouise , M. R. King , Biomicrofluidics 2011, 5, 024110. 21716809 10.1063/1.3596530 PMC3124519

[24] A. Aghakhani , A. Pena‐Francesch , U. Bozuyuk , H. Cetin , P. Wrede , M. Sitti , Sci. Adv. 2022, 8, abm5126. 10.1126/sciadv.abm5126 PMC8916727 35275716

[25] H. Bruus , Lab Chip 2012, 12, 1014. 22349937 10.1039/c2lc21068a

[26] D. Ahmed , C. Dillinger , A. Hong , B. J. Nelson , Adv. Mater. Technol. 2017, 2, 1700050.

[27] J.‐F. Louf , N. Bertin , B. Dollet , O. Stephan , P. Marmottant , Adv. Mater. Interfaces 2018, 5, 1800425.

[28] D. Ahmed , C. Dillinger , A. Hong , B. J. Nelson , Adv. Mater. Technol. 2017, 2, 1700050.

[29] A. Aghakhani , O. Yasa , P. Wrede , M. Sitti , Proc. Natl. Acad. Sci. USA 2020, 117, 3469. 32015114 10.1073/pnas.1920099117 PMC7035478

[30] L. Ren , N. Nama , J. M. Mcneill , F. Soto , Z. Yan , W. Liu , W. Wang , J. Wang , T. E. Mallouk , Sci. Adv. 2019, 5, 3084. 10.1126/sciadv.aax3084 PMC6814402 31692692

[31] F.‐W. Liu , S. K. Cho , Lab Chip 2021, 21, 355. 33305767 10.1039/d0lc00976h

[32] S. Mohanty , J. Zhang , J. M. Mcneill , T. Kuenen , F. P. Linde , J. Rouwkema , S. Misra , Sens. Actuators, B 2021, 347, 130589.

[33] D. Ahmed , M. Lu , A. Nourhani , P. E. Lammert , Z. Stratton , H. S. Muddana , V. H. Crespi , T. J. Huang , Sci. Rep. 2015, 5, 9744. 25993314 10.1038/srep09744 PMC4438614

[34] N. Bertin , T. A. Spelman , O. Stephan , L. Gredy , M. Bouriau , E. Lauga , P. Marmottant , Phys. Rev. Appl. 2015, 4, 064012.

[35] Q. Tang , F. Liang , L. Huang , P. Zhao , W. Wang , Biomed. Microdevices 2020, 22, 13. 31955256 10.1007/s10544-020-0470-1

[36] S. Zhang , Y. Wang , P. Onck , J. Den Toonder , Microfluid. Nanofluid. 2020, 24, 24.

[37] S. S. Sadhal , Lab Chip 2012, 12, 2771. 22776990 10.1039/c2lc40283a

[38] M. V. Patel , I. A. Nanayakkara , M. G. Simon , A. P. Lee , Lab Chip 2014, 14, 3860. 25124727 10.1039/c4lc00447g

[39] R. Das , Z. Ahmad , J. Nauruzbayeva , H. Mishra , Sci. Rep. 2020, 10, 7934. 32404874 10.1038/s41598-020-64345-1 PMC7221082

[40] X. Liu , H. Gu , H. Ding , X. Du , Z. He , L. Sun , J. Liao , P. Xiao , Z. Gu , Small 2019, 15, 1902360. 10.1002/smll.201902360 31305010

[41] S. Hu , X. Cao , T. Reddyhoff , D. Puhan , W. Huang , X. Shi , Z. Peng , D. Dini , ACS Appl. Mater. Interfaces 2019, 11, 20528. 31091076 10.1021/acsami.9b04020

[42] X. Liu , H. Gu , M. Wang , X. Du , B. Gao , A. Elbaz , L. Sun , J. Liao , P. Xiao , Z. Gu , Adv. Mater. 2018, 30, 1870157. 10.1002/adma.201800103 29603422

[43] C. Eckart , Phys. Rev. 1948, 73, 68.

[44] D. L. Miller , J. Acoust. Soc. Am. 1988, 84, 1378. 3198872 10.1121/1.396636

[45] K. J. Rao , F. Li , L. Meng , H. Zheng , F. Cai , W. Wang , Small 2015, 11, 2836. 25851515 10.1002/smll.201403621

[46] Z. Wu , T. Li , J. Li , W. Gao , T. Xu , C. Christianson , W. Gao , M. Galarnyk , Q. He , L. Zhang , J. Wang , ACS Nano 2014, 8, 12041. 25415461 10.1021/nn506200x PMC4386663

[47] X. Lu , Y. Wei , H. Ou , C. Zhao , L. Shi , W. Liu , Small 2021, 17, 2104516. 10.1002/smll.202104516 34608753

[48] Z. Huang , S. Zhao , M. Su , Q. Yang , Z. Li , Z. Cai , H. Zhao , X. Hu , H. Zhou , F. Li , J. Yang , Y. Wang , Y. Song , ACS Appl. Mater. Interfaces 2020, 12, 1757. 31818097 10.1021/acsami.9b15683

[49] Z. Wu , T. Li , J. Li , W. Gao , T. Xu , C. Christianson , W. Gao , M. Galarnyk , Q. He , L. Zhang , J. Wang , ACS Nano 2014, 8, 12041. 25415461 10.1021/nn506200x PMC4386663

[50] E. Lauga , Phys. Fluids 2007, 19, 061703.

[51] Z. Lin , X. Fan , M. Sun , C. Gao , Q. He , H. Xie , ACS Nano 2018, 12, 2539. 29443501 10.1021/acsnano.7b08344

[52] H.‐W. Huang , F. E. Uslu , P. Katsamba , E. Lauga , M. S. Sakar , B. J. Nelson , Sci. Adv. 2019, 5, aau1532. 10.1126/sciadv.aau1532 PMC6357760 30746446

[53] U. Bozuyuk , A. Aghakhani , Y. Alapan , M. Yunusa , P. Wrede , M. Sitti , Nat. Commun. 2022, 13, 6289. 36271078 10.1038/s41467-022-34023-z PMC9586970

[54] S. Mohanty , A. Paul , P. M. Matos , J. Zhang , J. Sikorski , S. Misra , Small 2022, 18, 2105829. 10.1002/smll.202105829 34889051

[55] A. A. Doinikov , A. Bouakaz , J. Fluid Mech. 2014, 742, 425.

[56] P. Spurný , J. Oberst , D. Heinlein , Nature 2003, 423, 151. 12736679 10.1038/nature01592

[57] M. Shehadul Islam , A. Aryasomayajula , P. Selvaganapathy , Micromachines 2017, 8, 83.

[58] G. Cai , L. Xue , H. Zhang , J. Lin , Micromachines 2017, 8, 274. 30400464 10.3390/mi8090274 PMC6189760

[59] C. F. G. C. Geraldes , S. Laurent , Contrast Media Mol. Imaging 2009, 4, 1. 19156706 10.1002/cmmi.265

[60] J. Wu , G. Du , J. Acoust. Soc. Am. 1997, 101, 1899.

[61] T. G. Leighton , The Acoustic Bubble, Elsevier, Amsterdam, Netherlands 1994.

[62] M. S. Longuet‐Higgins , Proc. R. Soc. London, Ser. A 1998, 454, 725.

[63] T. A. Spelman , E. Lauga , J. Eng. Math. 2017, 105, 31.

[64] N. Bertin , T. A. Spelman , T. Combriat , H. Hue , O. Stéphan , E. Lauga , P. Marmottant , Lab Chip 2017, 17, 1515. 28374878 10.1039/c7lc00240h

