# Supplementary material for: Acoustic Streaming‐Induced Multimodal Locomotion of Bubble‐Based Microrobots
Source: Adv Sci (Weinh). 2023 Oct 26;10(35):2304233. doi: 10.1002/advs.202304233 (PMC10724404; doi:10.1002/advs.202304233)
Supplement: Supplementary file 1 — Supporting Information [file ADVS-10-2304233-s004.pdf]

ADVANCED SCIENCE

Open Access

Supporting Information

for *Adv. Sci.*, DOI 10.1002/advs.202304233

Acoustic Streaming-Induced Multimodal Locomotion of Bubble-Based Microrobots

*Nima Mahkam, Amirreza Aghakhani, Devin Sheehan, Gaurav Gardi, Robert Katzschmann and Metin Sitti**

Supporting Information

Acoustic streaming-induced multimodal locomotion of bubble-based microrobots

Nima Mahkam, Amirreza Aghakhani, Devin Sheehan, Gaurav Gardi, Robert Katzschmann, Metin Sitti

Correspondence to: Metin Sitti
Email: sitti@is.mpg.de

This PDF file includes:

Supporting text
Figures S1 to S23
Tables S1 and S2
Legends for Movies S1 to S10
SI References

Other supporting materials for this manuscript include the following:

Movies S1 to S10

Supporting Information Text

S1. Resonance frequencies and pressure map

The Rayleigh-Plasset[1] equation is as follows:

$$f = \sqrt{\frac{1}{4\rho\pi^2 r^2}\left(3k\left(p + \frac{2\sigma}{r}\right) - \frac{2\sigma}{r}\right)}, \quad (1)$$

in which, ρ is the density of the liquid [$\frac{\mathrm{kg}}{\mathrm{m}^3}$], σ is the surface tension of solution [$\frac{\mathrm{N}}{\mathrm{m}}$], k is the polytopic exponent for the bubble,[2] p is the atmospheric pressure [Pa], and $r = \frac{D_c}{2}$ is the radius of the bubble.

The resonance frequency of the trapped bubble inside the cavity is calculated using Equation (1). Considering the bubble diameter of D_C = 30 mm the resonance frequency of the tapped bubble is 218 kHz. However, the actuation frequencies of the experiments within this study are in the range of 70 to 270 kHz. It's important to note that the actuation at resonance only enhances the flow velocities through larger air-liquid interface oscillation amplitudes. Different locomotion modes appear due to diverse flow patterns at different frequencies. Also, the stream velocities would affect the bulk flows around the fluid. Yet, in our robot design, different locomotion modes are more feasible in the 70 to 150 kHz range. This can be attributed to the power outcome of the piezo transducer used in this study that provides higher pressure amplitudes within the range mentioned above. We measured the pressure within the medium inside the chamber using an acoustic hydrophone at different frequencies and chamber to piezoelectric distances. The pressure measurements show a considerable drop in the pressure values at actuation frequencies f = 150 kHz and f = 200 kHz and intuitively as the distance between the chamber and the piezo is increased.

Figure S7 provides a detailed pressure distribution map within the chamber, revealing variations across different frequencies and power amplitudes. The experimental setup, depicted in Figure S22, comprises a glass structure, a piezoelectric transducer, and the chamber. The chamber plays a vital role in maintaining a closed system, ensuring the accuracy of measurements. To prevent the formation of standing waves that could interfere with the results, a PDMS cab is carefully positioned on top of the chamber, effectively minimizing unwanted reflections (cab dia. 14 mm). The acoustic mechanism relies on the generation of traveling waves within the chamber, achieved through the utilization of surface acoustic waves produced by the attached piezoelectric transducer located in close proximity to the chamber.[3] The distinct pressure regions observed in the map can be attributed to the reflection of waves at the edges of the glass, resulting in localized variations in the pressure distribution.

S2. Bubble stability

We performed a set of experiments to investigate the bubble's stability with two conditions: (I) passive stability, where the microrobots were immersed inside a medium and stored for 7 days, (II) active stability, where the microrobots were observed during continuous acoustic actuation for up to 10 hours. Passive stability test results showed no dissolved bubbles within this time frame (Figure S8). For the active stability analyses, three sets of analyses were performed to determine the bubble stability at medium to high flow rates at V = 3, 5, and 7 Vpp. The spherical cavity design helps to improve the bubble stability performance. After 10 hours of continuous oscillation, 91%, 84%, and 54% of the bubbles survived at V = 3, 5, and 7 Vpp actuation voltages, respectively (the actuation frequency is in range of 100 kHz $\leq f \leq$ 120 kHz, where high flow rates are observed). At piezoelectric input voltage of V = 3 and 5 Vpp the unstable bubbles started dissolving at very early stages of actuation (t < 30 minutes), which are related to the print uncertainties of every individual microrobot (see Movie S9, and Figure S9-S11).

S3. Radiation and streaming force

The radiation force acting on the rigid shell scales like $F_r \sim -< \Delta V(t)\Delta p(t) >$ which is a time-averaged forcing component (structure size << wavelength of the acoustic wave), in which $\Delta V(t)$ is the bubble volume change, and $\Delta p(t) \sim p_{in} f / c_l$ is the pressure gradient. In this equation, f is the wave frequency, p_{in} instant pressure value, and c_l is the speed of sound inside the chamber. On the other hand, non-linear streaming forces scale like $F_{st} \sim \varepsilon_i^2 p_l R_o^4 f^2$, in which, R_0 and ε_i are the radius of the non-oscillating bubble and pulsation amplitude of every orifice, respectively.[4] The ratio of the acoustic streaming force to the acoustic radiation force lies in the range of $\frac{F_{st}}{F_r} \sim 5 - 45,$ indicating the dominancy of the streaming force for low amplitude sound waves at microscale over the radiation force.[5,6]

S4. Bjerknes force

Air bubbles are trapped in the cavities after immerging the microrobots inside the medium. Under the action of the sound wave, and due to the primary radiation forces[7] of the oscillatory bubble, microrobots flip, where the distance of all orifices and the substrate are minimal. The primary Bjerknes force of an oscillatory bubble with a radius R can be calculated using the following equation:

$$\boldsymbol{F_{PB}} = -\frac{4}{3}\pi R^3 (\frac{dp}{dx_i}), \quad (2)$$

in which p expresses the instantaneous pressure at the position of the microrobot.

The secondary Bjerknes force ($\boldsymbol{F_{SB}}$) of an oscillatory bubble with a radius R on similar sized bubble in position **X** can be calculated using the following equation [8]:

$$\boldsymbol{F_{SB}} = \frac{2\pi\rho R_0^4\omega^2\delta^2\boldsymbol{X}}{r^3}, \qquad (3)$$

in which ω expresses radial oscillation frequency, δ is the radial oscillation amplitude. R_0 is the bubble radius, and **X** is the relative position of the second bubble.

Microrobot with only two 13 μm in diameter side orifices donated as MrS – orifices are parallel to the substrate after the flipping stage.

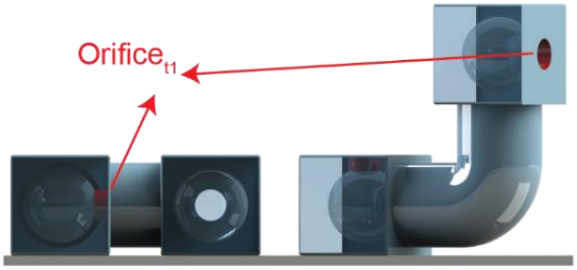

Figure S1. MrS with two 13 μm in-diameter orifices at the side of the base.

The microrobot dimensions used in this study are as follows:

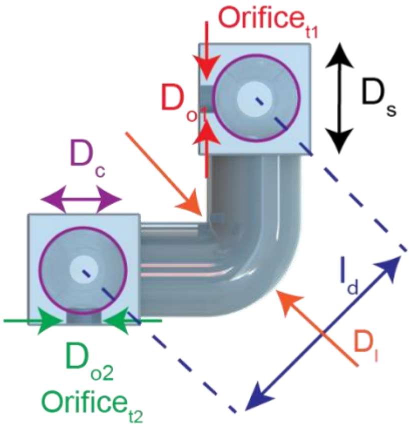

Figure S2. Schematic of the L-shaped acoustically-powered microrobot.

Table S1. Microrobot dimensions

D_c	D_s	D_{o1}	D_{o2}	l_d	D_l
30 μm	40 μm	13 μm	11 μm	85 μm	34 μm

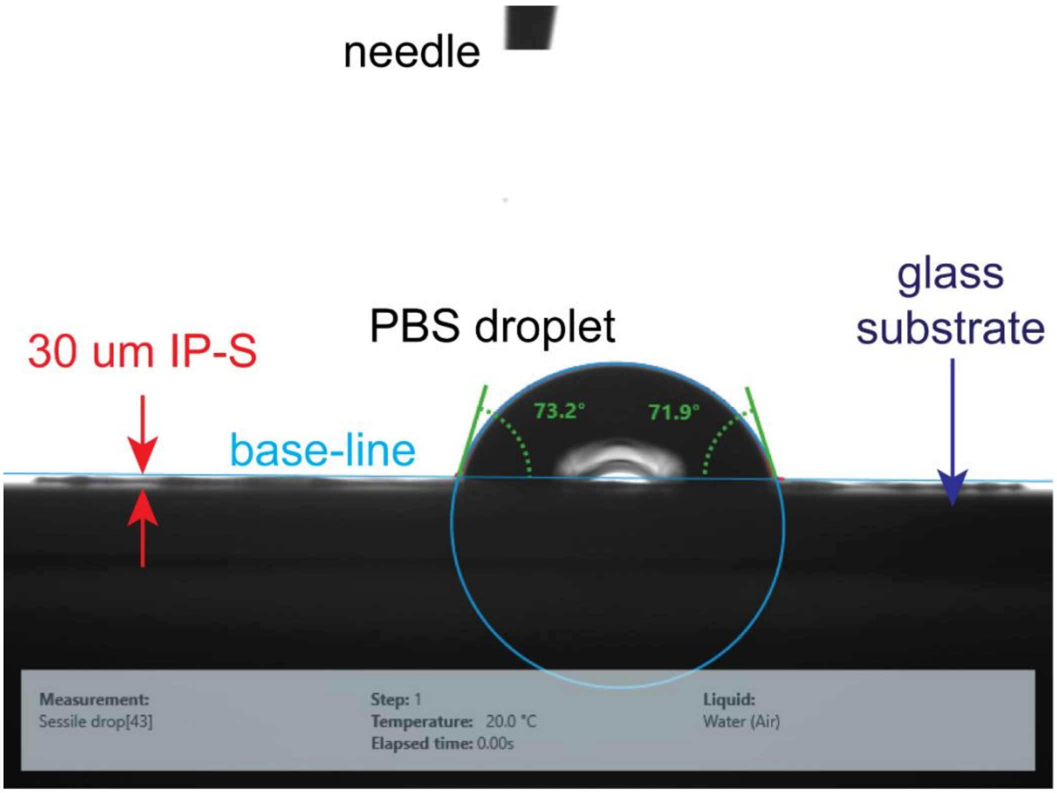

Figure S3. Contact angle measurement of the printed IP-S resin with PBS droplet. A 10 mm cylinder, with a thickness of 30 micrometers, is printed onto a glass substrate using IP-S resin for contact angle measurements.

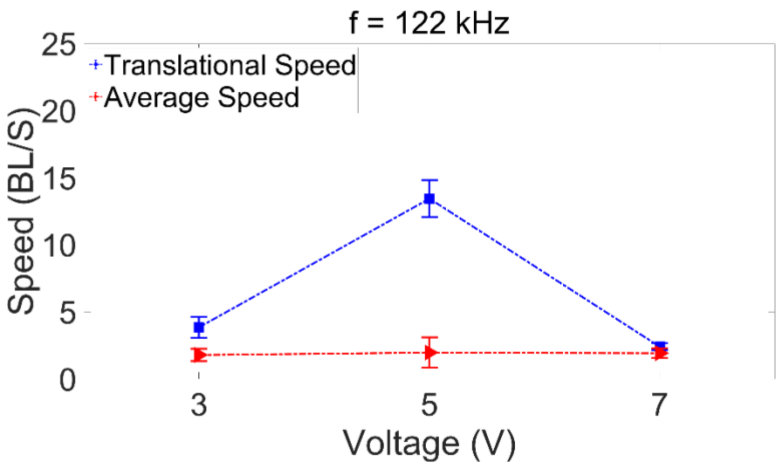

Figure S4. The velocity of the microrobot with four orifices (MrSB) at a sound wave frequency of f = 122 kHz. The error bars show the standard deviation of three different tests.

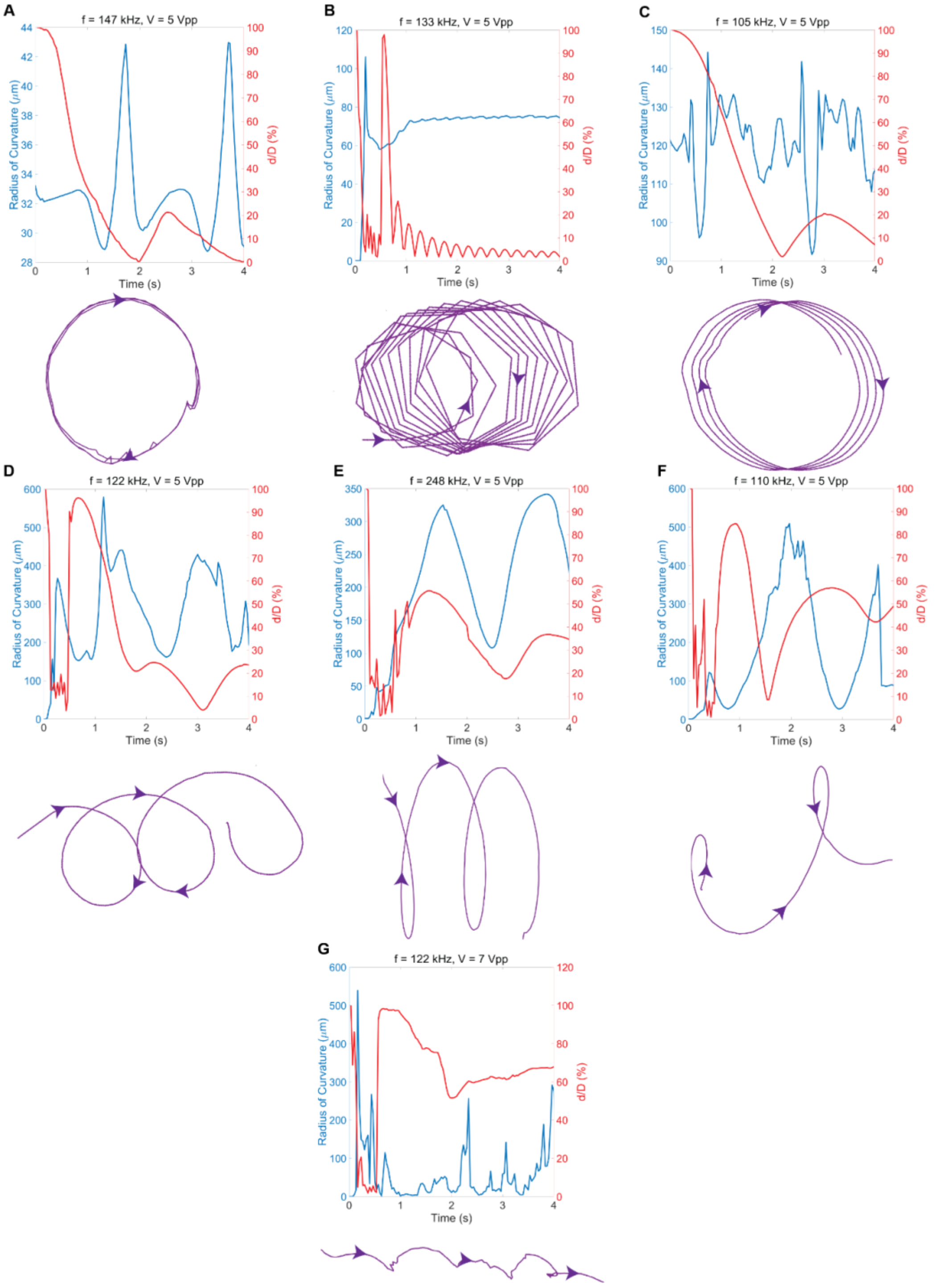

Figure S5. *RoC*, $\frac{d}{D}$, and the trajectory of the microrobots under different sound wave frequencies for MrSB. From **(A-G)** the $\frac{d}{D}$ increases that corresponds to a transition of the locomotion mode from S to R, TR and finally T mode gradually. The locomotion is both power and frequency dependent.

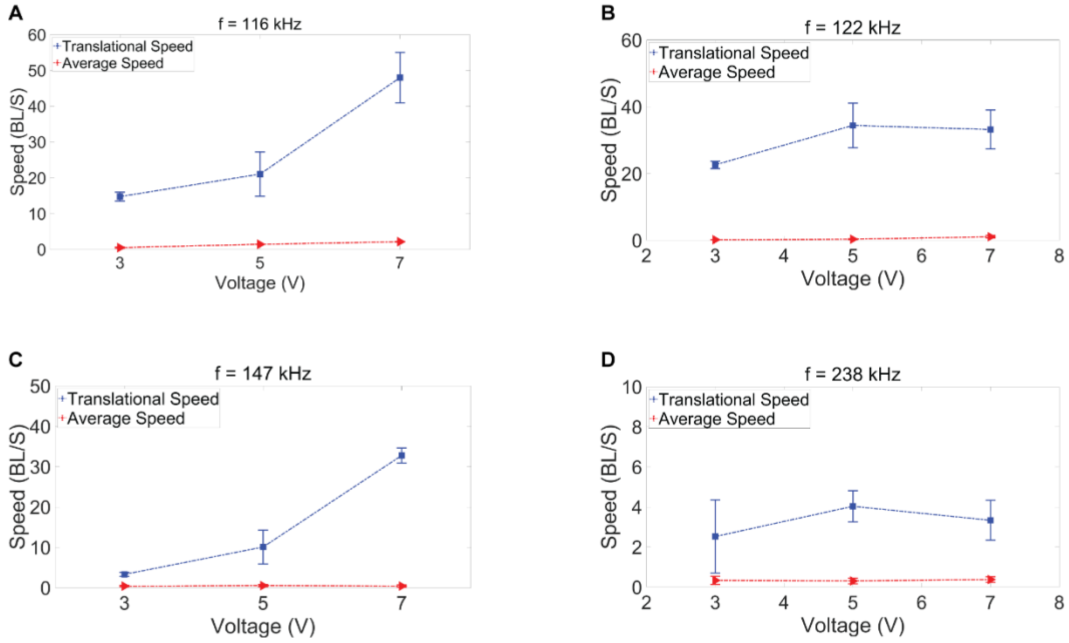

Figure S6. Translational and average speed of MrB at different voltage inputs and sound wave frequencies. (**A**) f = 116 kHz, (**B**) f = 122 kHz, (**C**) f = 147 kHz, and (**D**) f = 238 kHz. The error bars show the standard deviation of three different tests.

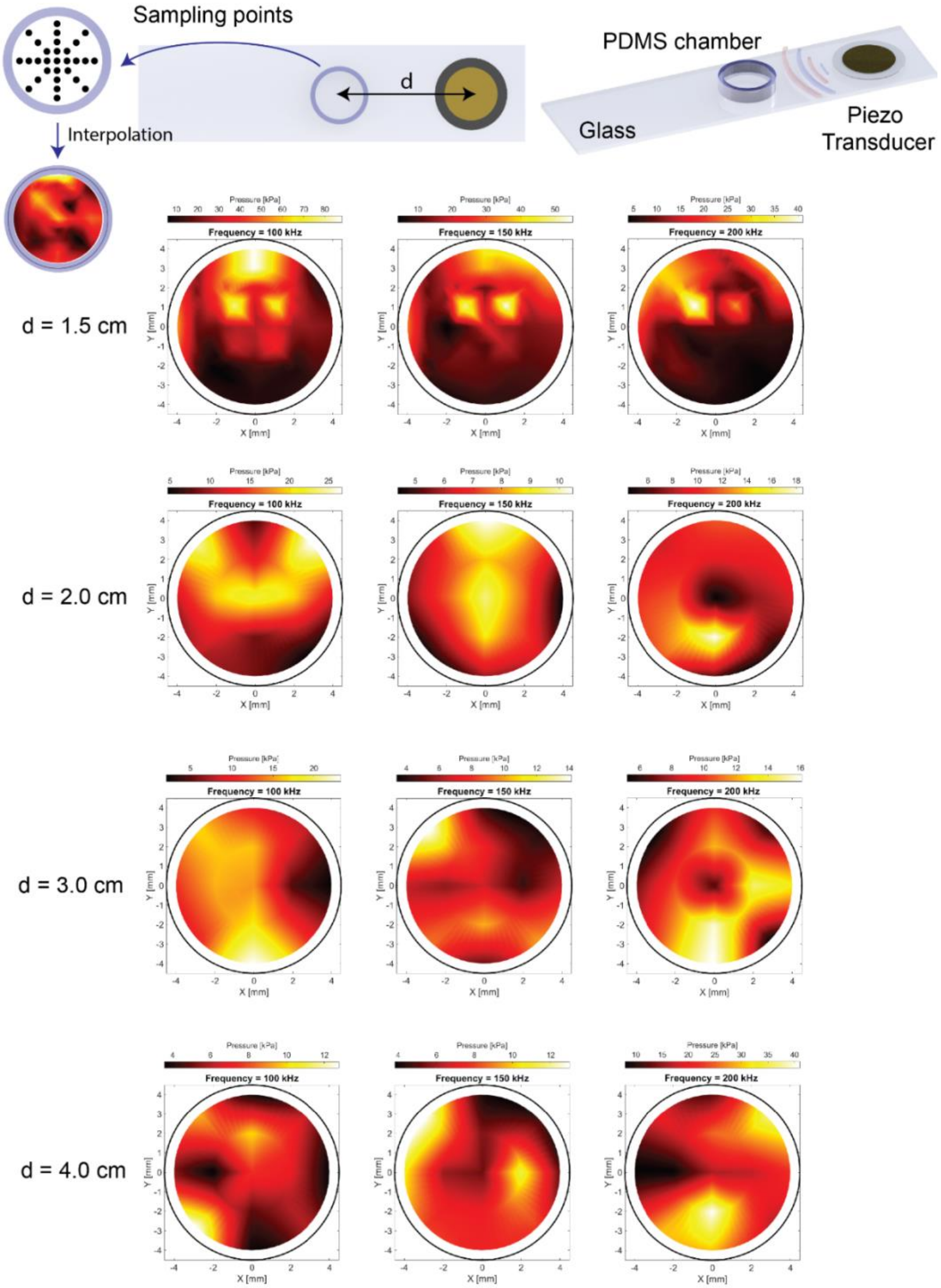

Figure S7. Pressure map of the chamber at 100 μm away from the glass substrate. The pressure values are measured for actuation frequency of f = 100 kHz, 150 kHz, and 200 kHz and V = 5 V_{pp}. d shows distance between the center of the piezoelectric transducer and the chamber's origin.

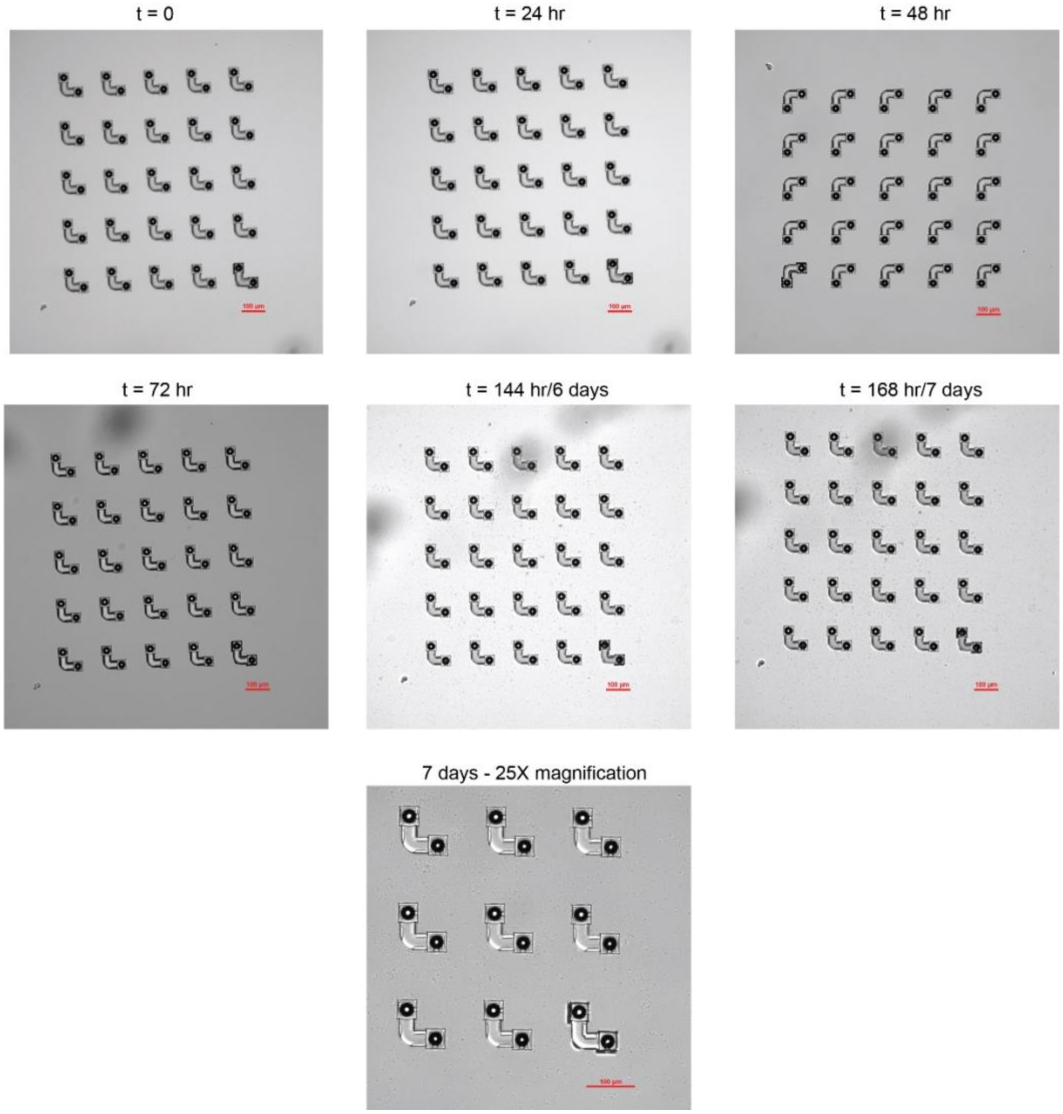

Figure S8. Bubble passive stability tests. Microrobots are immersed inside chamber filled with PBS and observed for 7 days. After 7 days, the bubbles remain stable and do not diffuse.

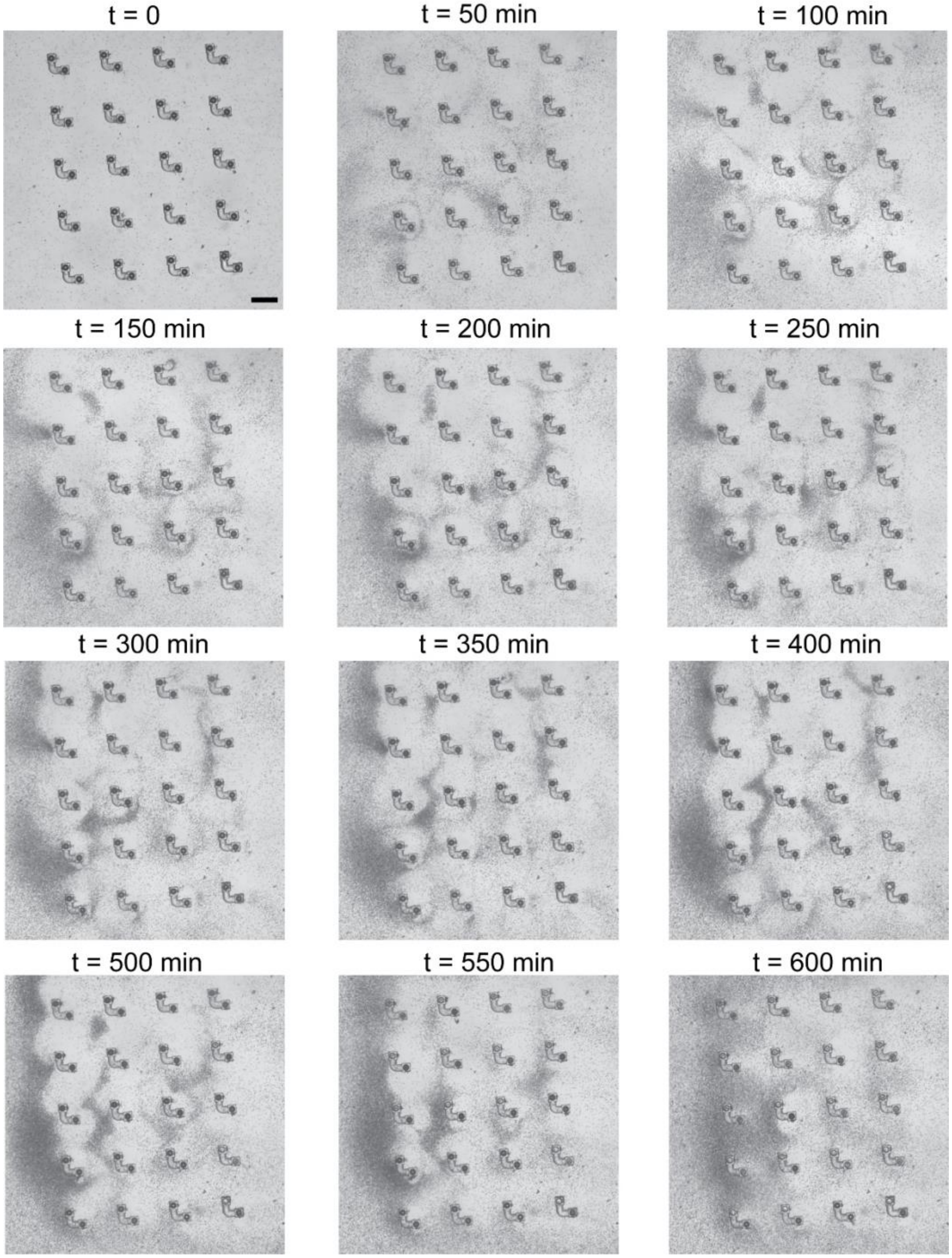

Figure S9. Bubble active stability tests. The continuous sound wave at f = 126 kHz and V = 3 Vpp is transmitted to the chamber while the robots are anchored to the substrate. After 10 hours of operation, a viability of 75% is attained.

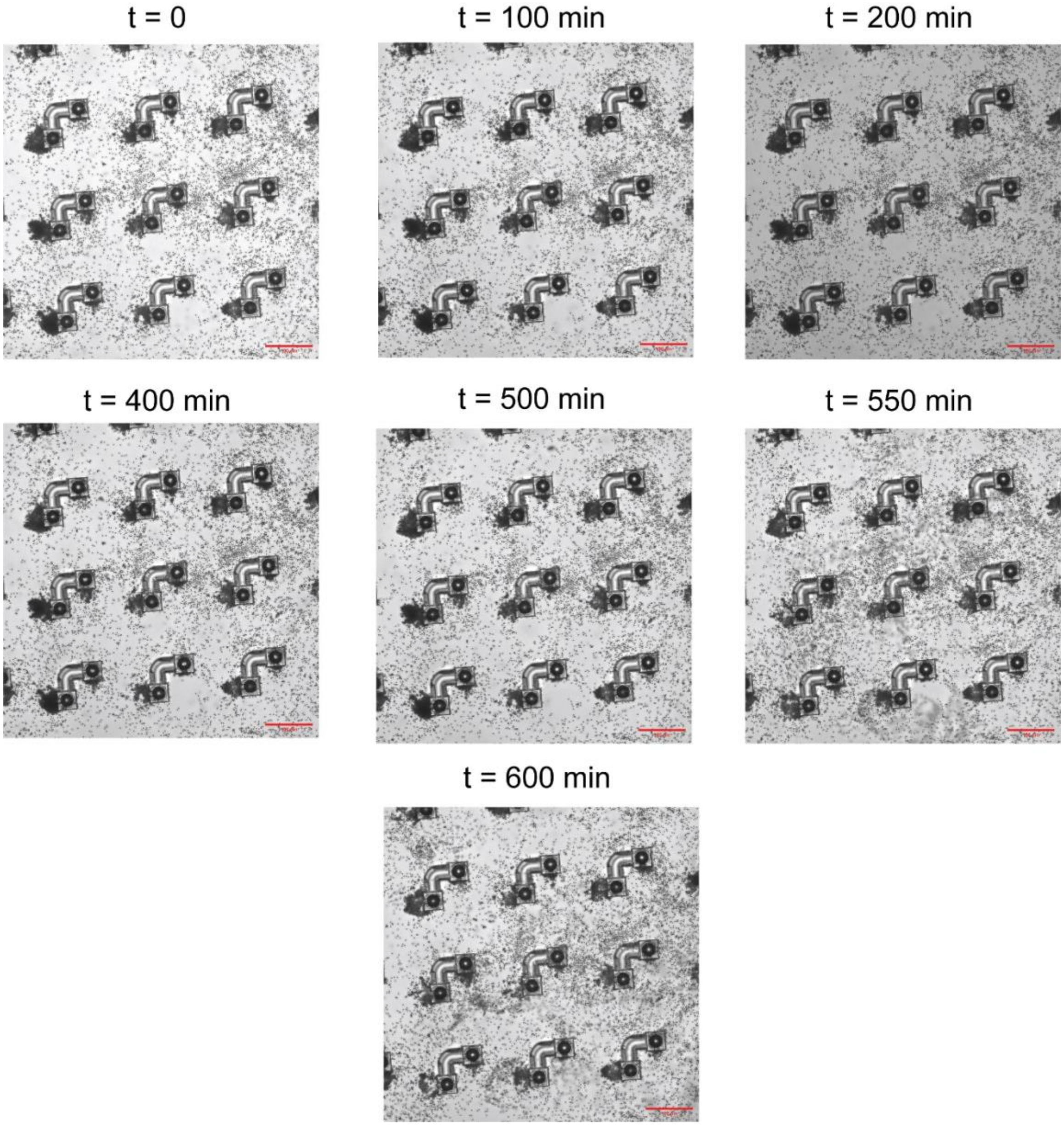

Figure S10. Bubble active stability tests. The continuous sound wave at *f* = 180 kHz and *V* = 3 Vpp is transmitted to the chamber while the robots are anchored to the substrate. After 10 hours of operation, a viability of 100% is attained.

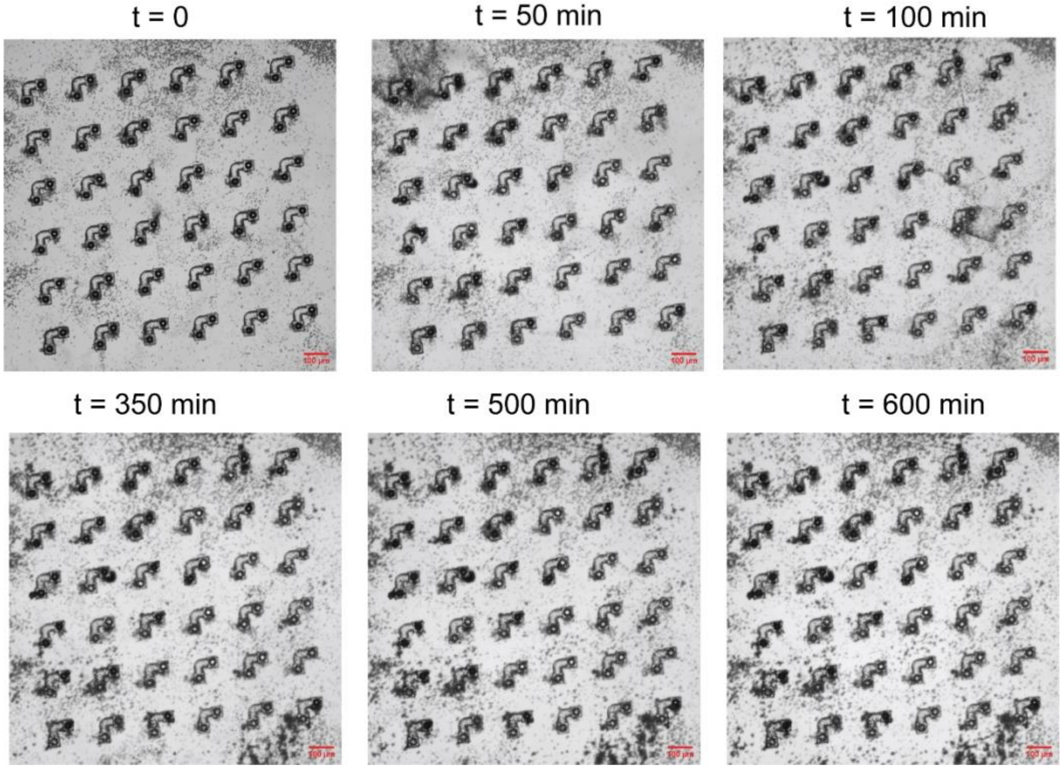

Figure S11. Bubble active stability tests. The continuous sound wave at *f* = 147 kHz and *V* = 7 Vpp is transmitted to the chamber while the robots are anchored to the substrate. After 10 hours of operation, a viability of 51% is attained.

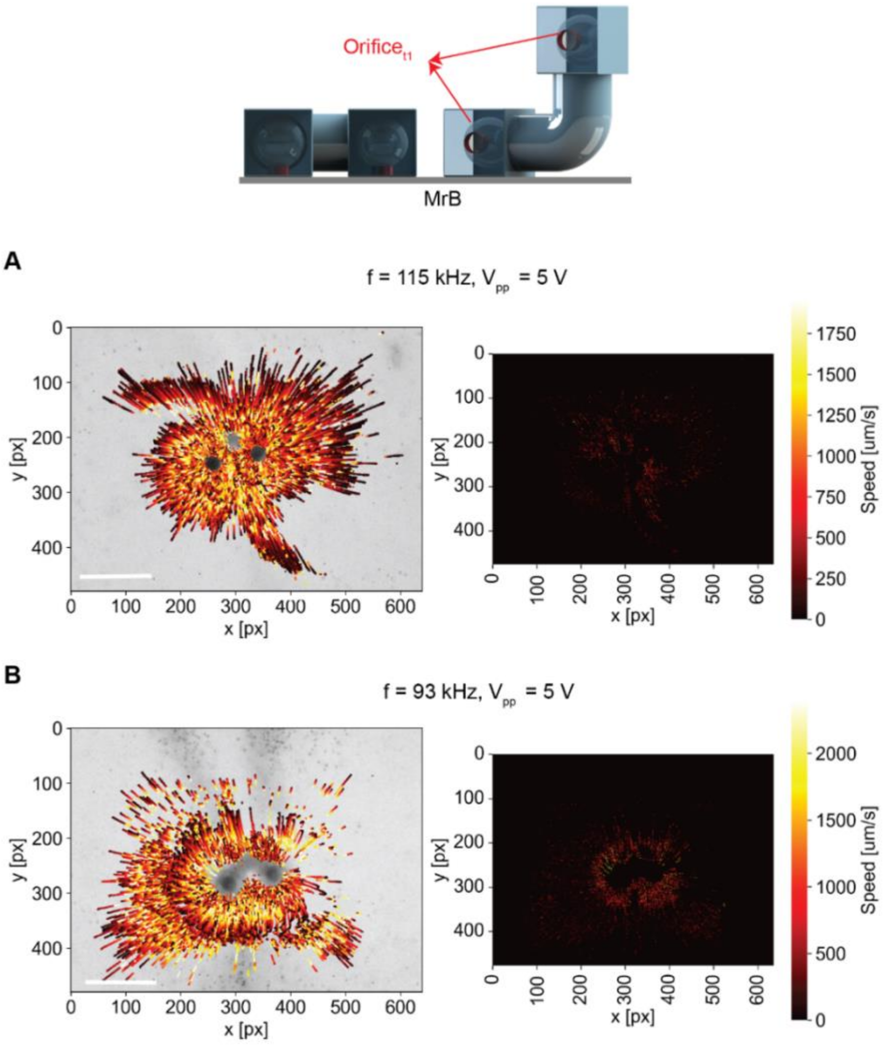

Figure S12. Acoustic microstreaming of two 30 μm oscillating bubbles with only two 13 μm bottom orifices (MrB). Orifices are facing to the positive *z* direction, i.e., orifices facing upward. Scale bars: 100 μm.

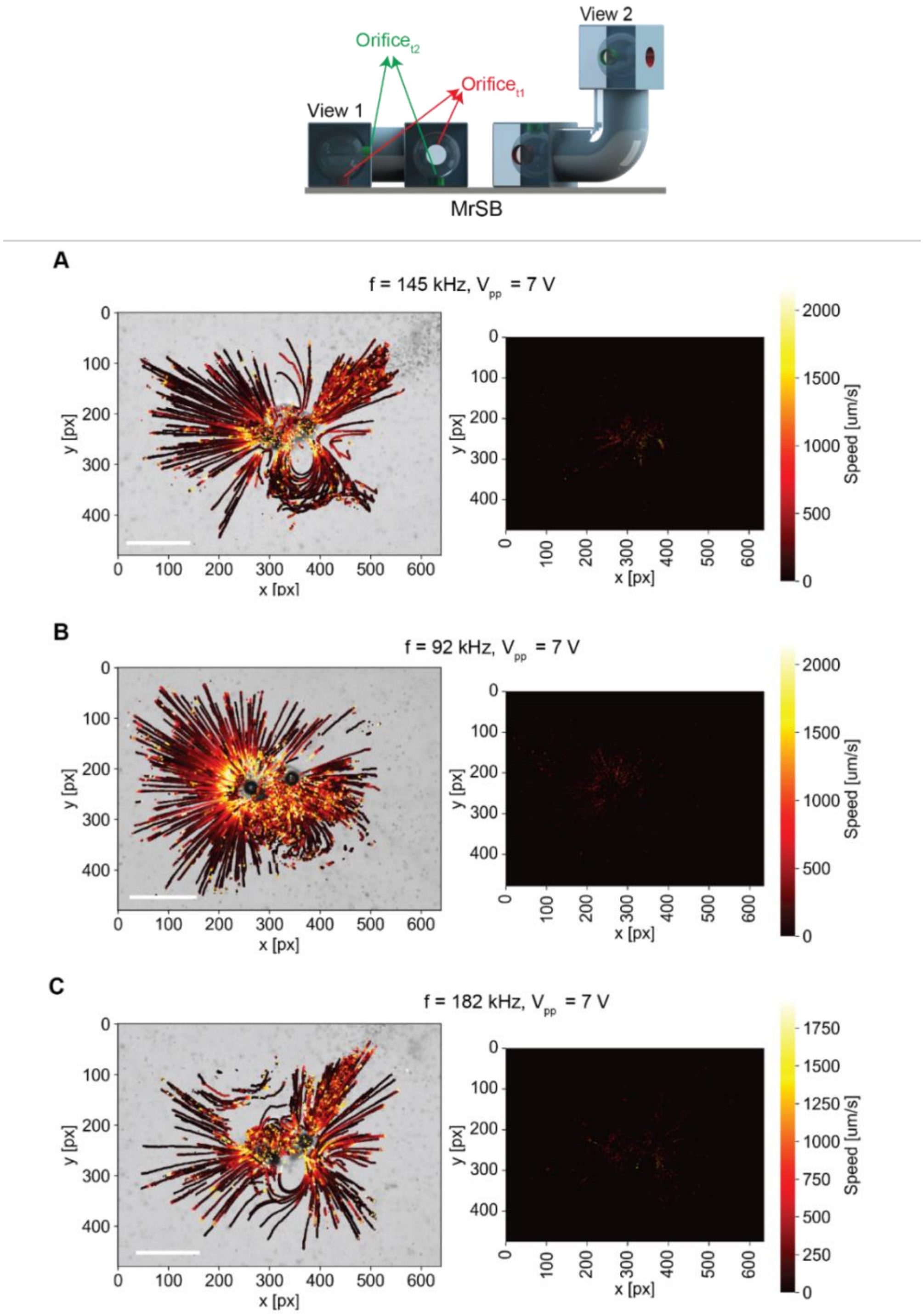

Figure S13. Bulk streaming of two 30 μm bubbles with four orifices at the side and bottom (MrSB). The bottom orifices are facing the positive *z* direction (i.e., facing upward). The interaction of two oscillating bubbles results in combination of closed curved flows, vortices, and jets away and toward the microstructure. Scale bars: 100 μm.

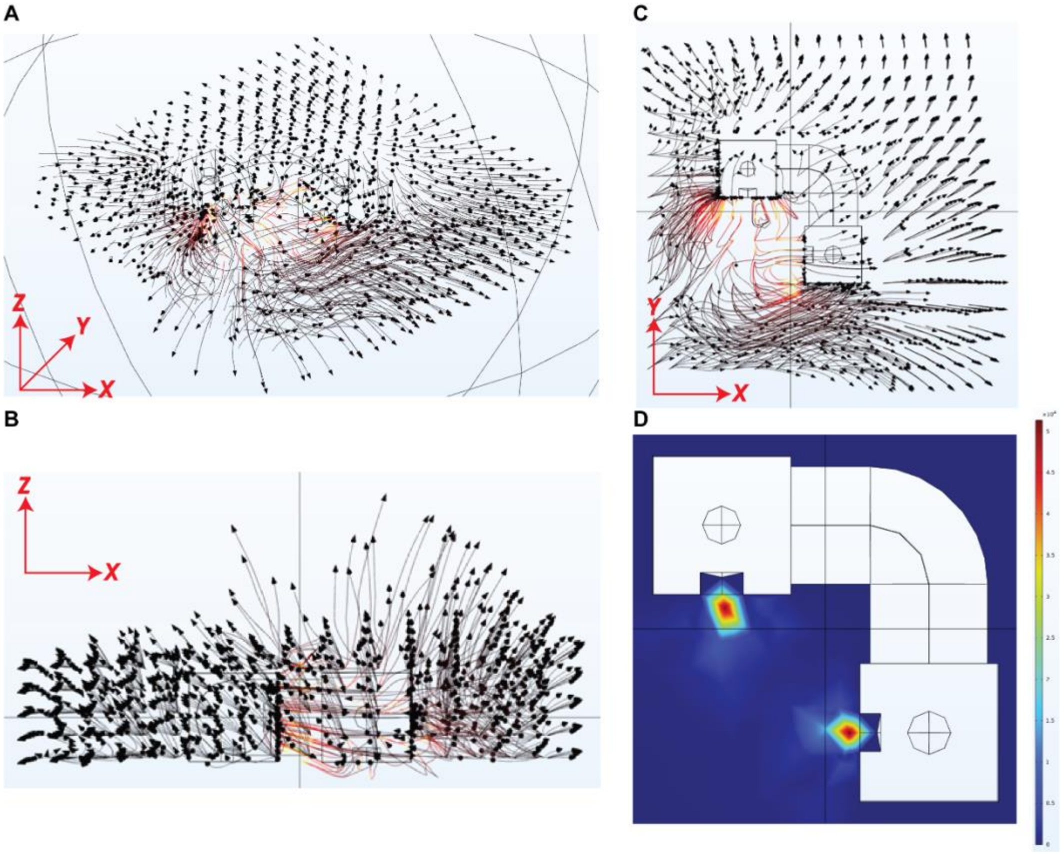

Figure S14. Simulated 3D flows of an L-shaped robot at f = 92 kHz. Acoustic streaming of two coupled bubbles with (**A**) 1-point perspective, (**B**) XZ-view, and (**C**) XY-view. (**D**) Flow rates of two side-nozzles in XY-plane.

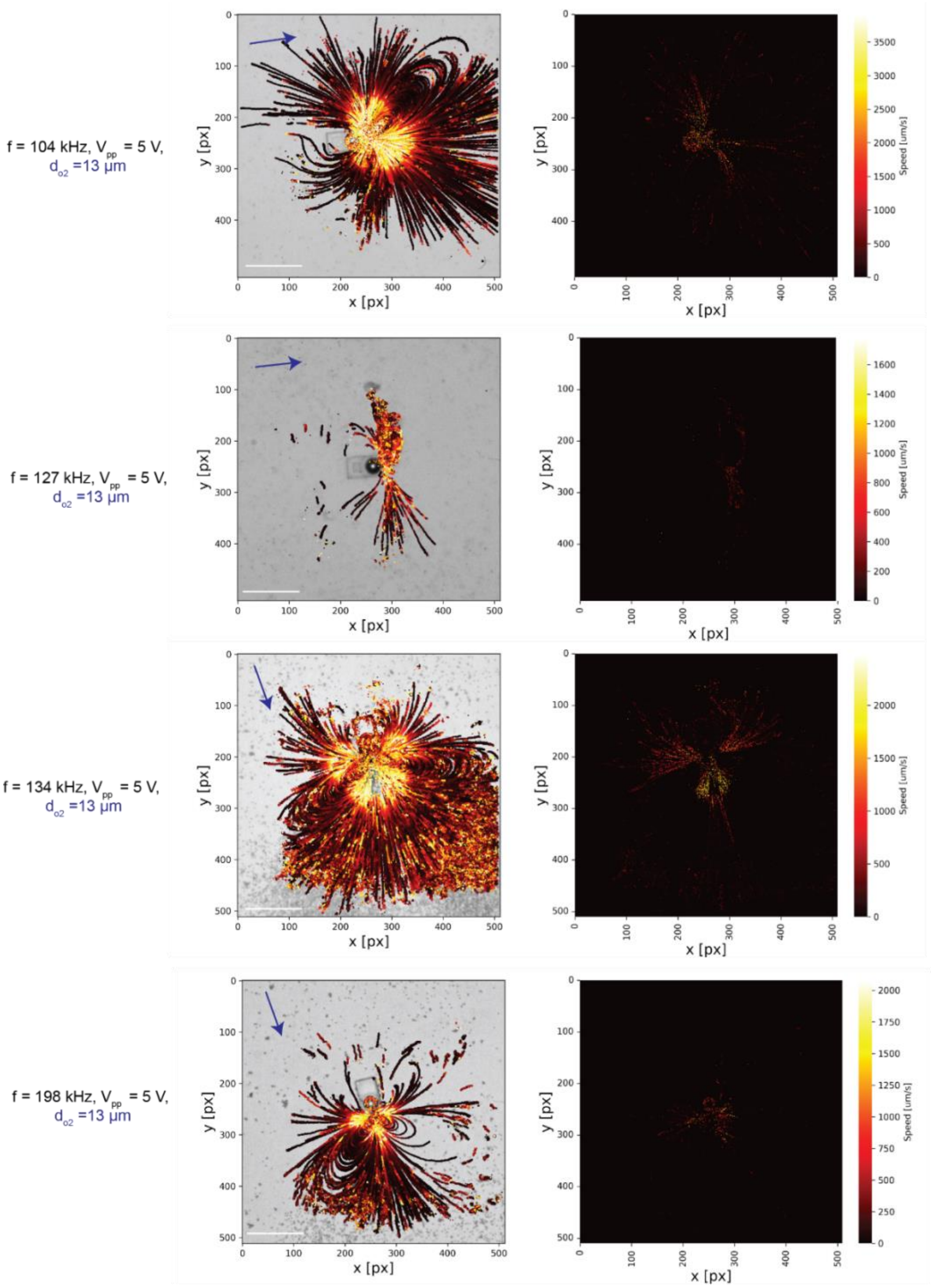

Figure S15. Acoustic microstreaming of a 30 μm bubble with a single 13 μm side nozzle. Particle trajectories and instantaneous speed of the particle (left), and spatial mean speed of the particles passing through the $\boldsymbol{i^{th}}$ and $\boldsymbol{j^{th}}$ pixel for 5 s (right). Arrows show the direction of the orifice. Scale bars: 100 μm.

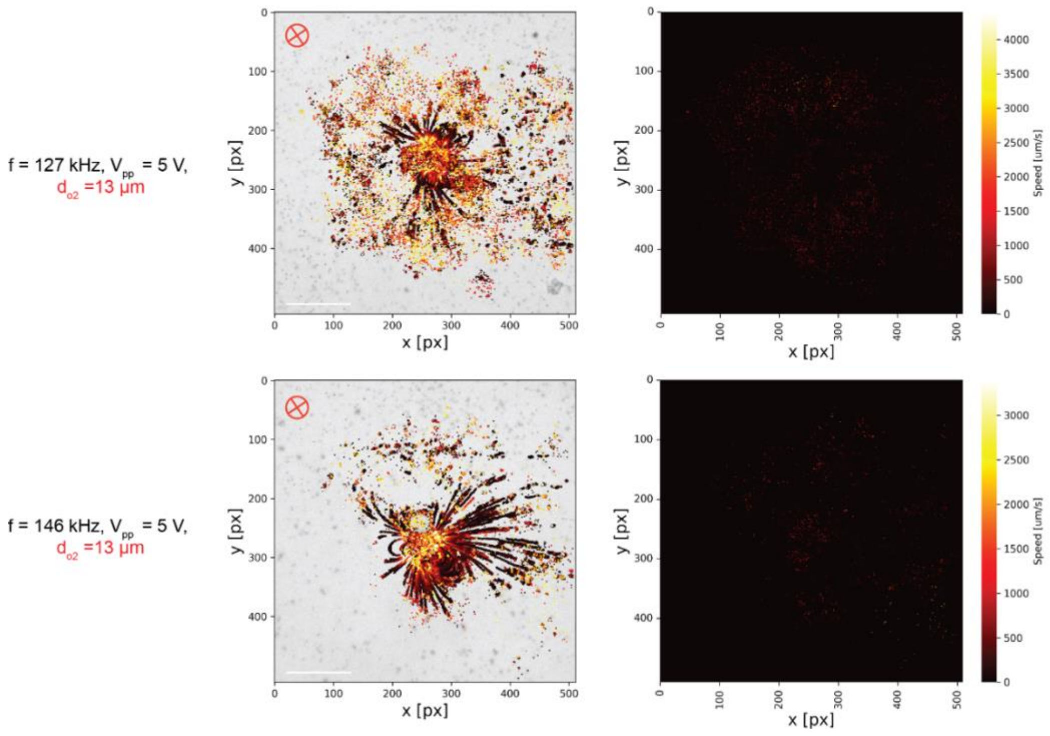

Figure S16. A single 13 μm nozzle and 30 μm bubble is positioned 5 μm away from a rigid wall facing the substrate. Particle trajectories and instantaneous speed of the particle (left), and spatial mean speed of the particles passing through the ith and j^{th} pixel for 5 s (right). Arrows show the direction of the orifice. Scale bars: 100 μm.

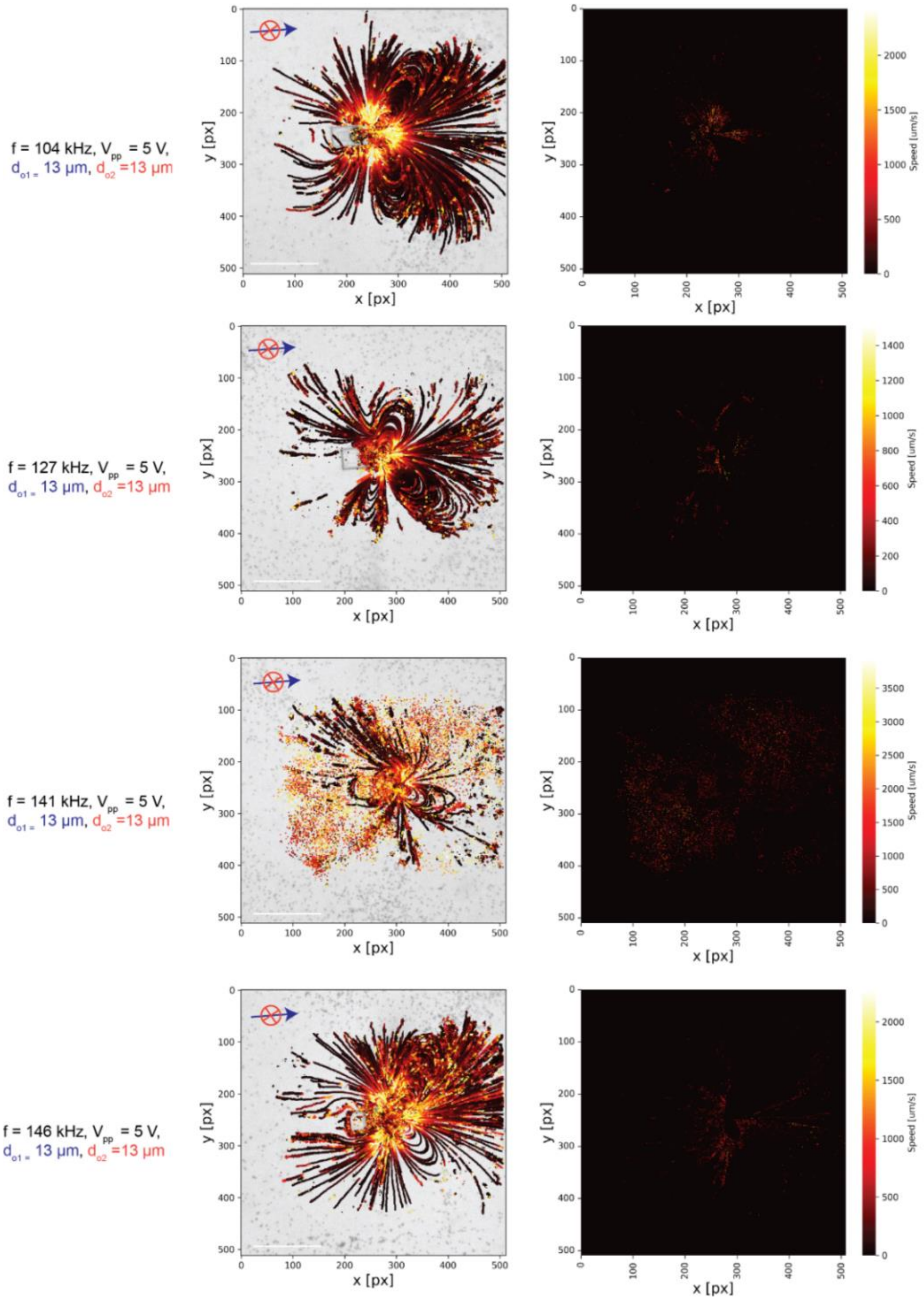

Figure S17. Acoustic microstreaming of a 30 μm trapped bubble with two 13 μm nozzles. One nozzle facing the substrate at 5 μm away from the rigid wall, the other nozzle parallel to the substrate at $\boldsymbol{l_{db}}$ = 25 μm. Particle trajectories and instantaneous speed of the particle (left), and spatial mean speed of the particles passing through the $\boldsymbol{i^{th}}$ and $\boldsymbol{j^{th}}$ pixel for 5 s (right). Arrows show the direction of the orifice. Scale bars: 100 μm.

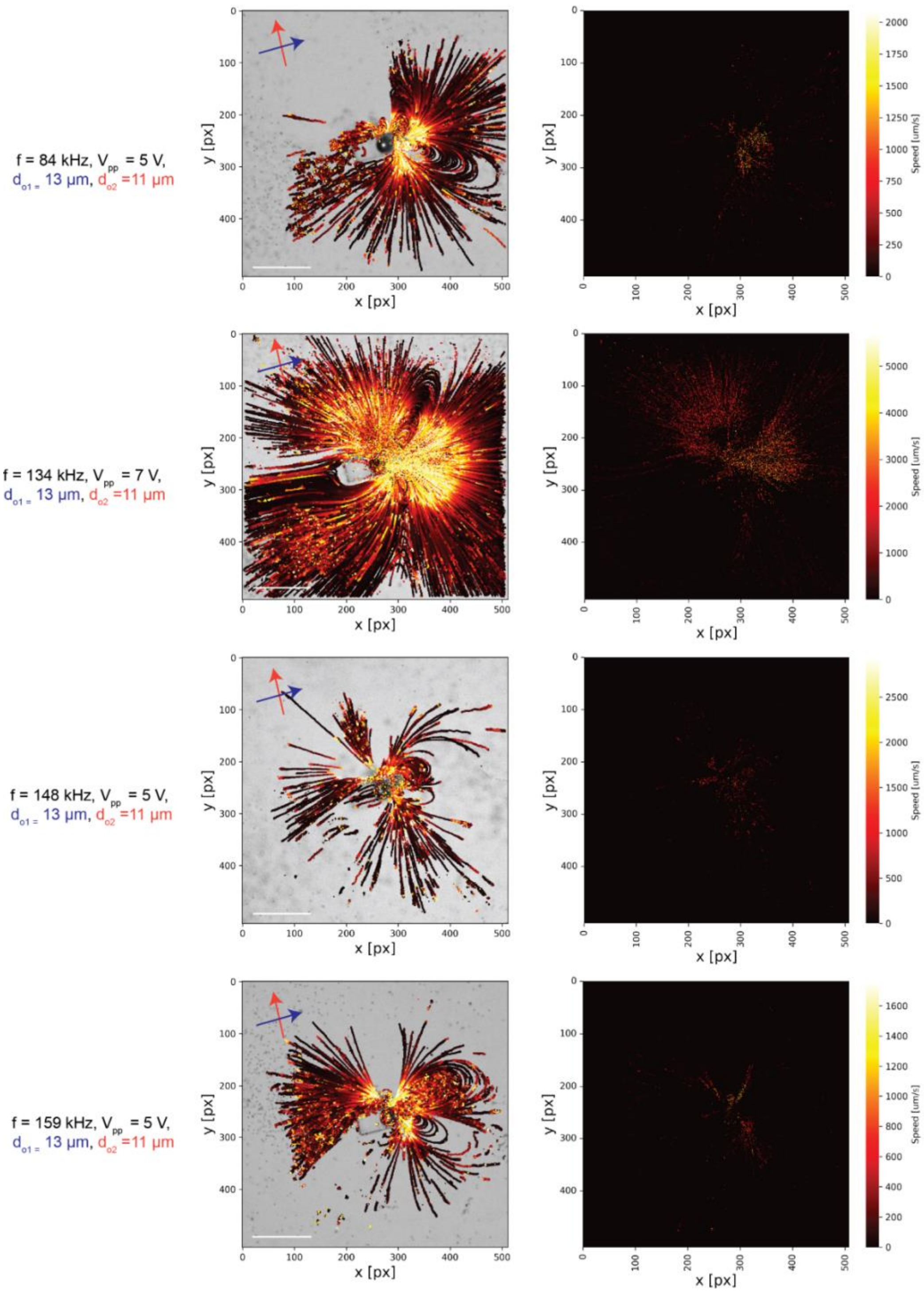

Figure S18. Acoustic microstreaming of a 30 μm bubble with two 11 μm and 13 μm side orifices. Particle trajectories and instantaneous speed of the particle (left), and spatial mean speed of the particles passing through the $\boldsymbol{i^{th}}$ and $\boldsymbol{j^{th}}$ pixel for 5 s (right). Arrows show the direction of the orifice. Scale bars: 100 μm.

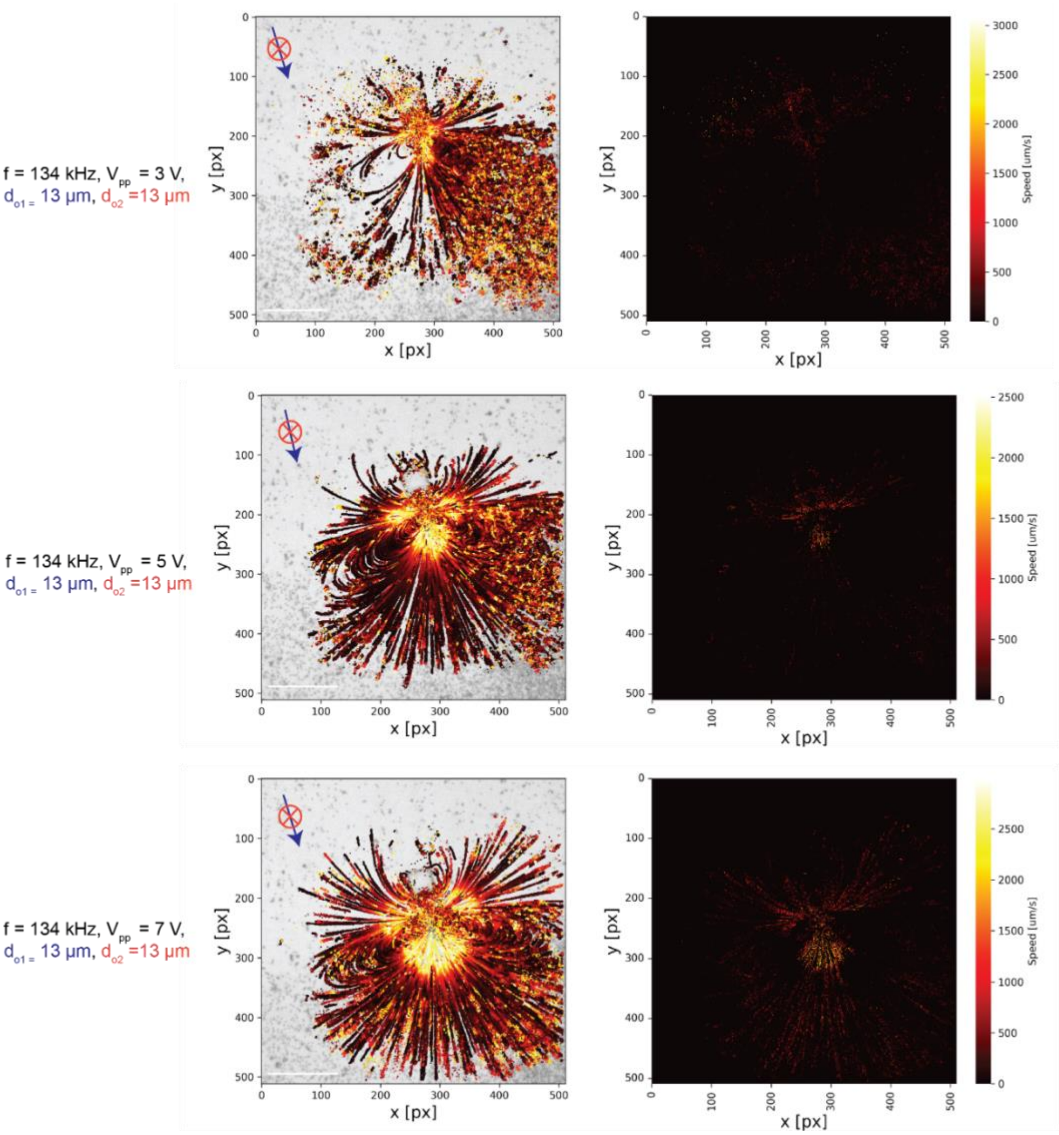

Figure S19. The effect of the increased voltage on the acoustic microstreaming of a 30 μm microbubble with 13 μm orifices. The $\boldsymbol{l_{db}}$ for the bottom and the side orifice are 5 μm and 25 μm, respectively. Sound wave frequency is f = 134 kHz, and power indicates the input voltage for the piezoelectric transducer attached to the glass at the vicinity of the chamber. Arrows show the direction of the orifice. Scale bars: 100 μm.

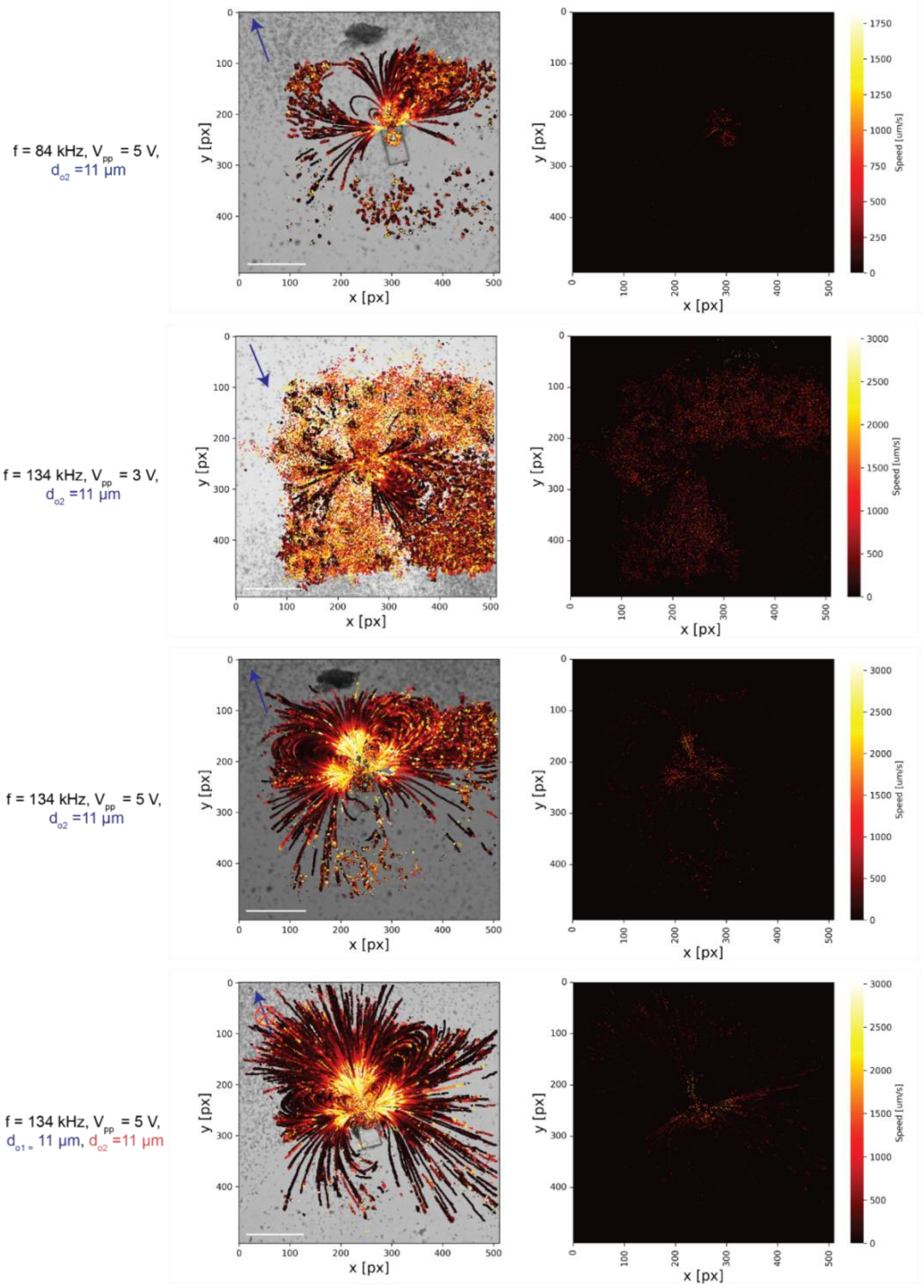

Figure S20. Acoustic microstreaming of a 30 μm microbubble with 11 μm side orifices. Particle trajectories and instantaneous speed of the particle (left), and spatial mean speed of the particles passing through the ith and j^{th} pixel for 5 s (right). Arrows show the direction of the orifice. Scale bars: 100 μm.

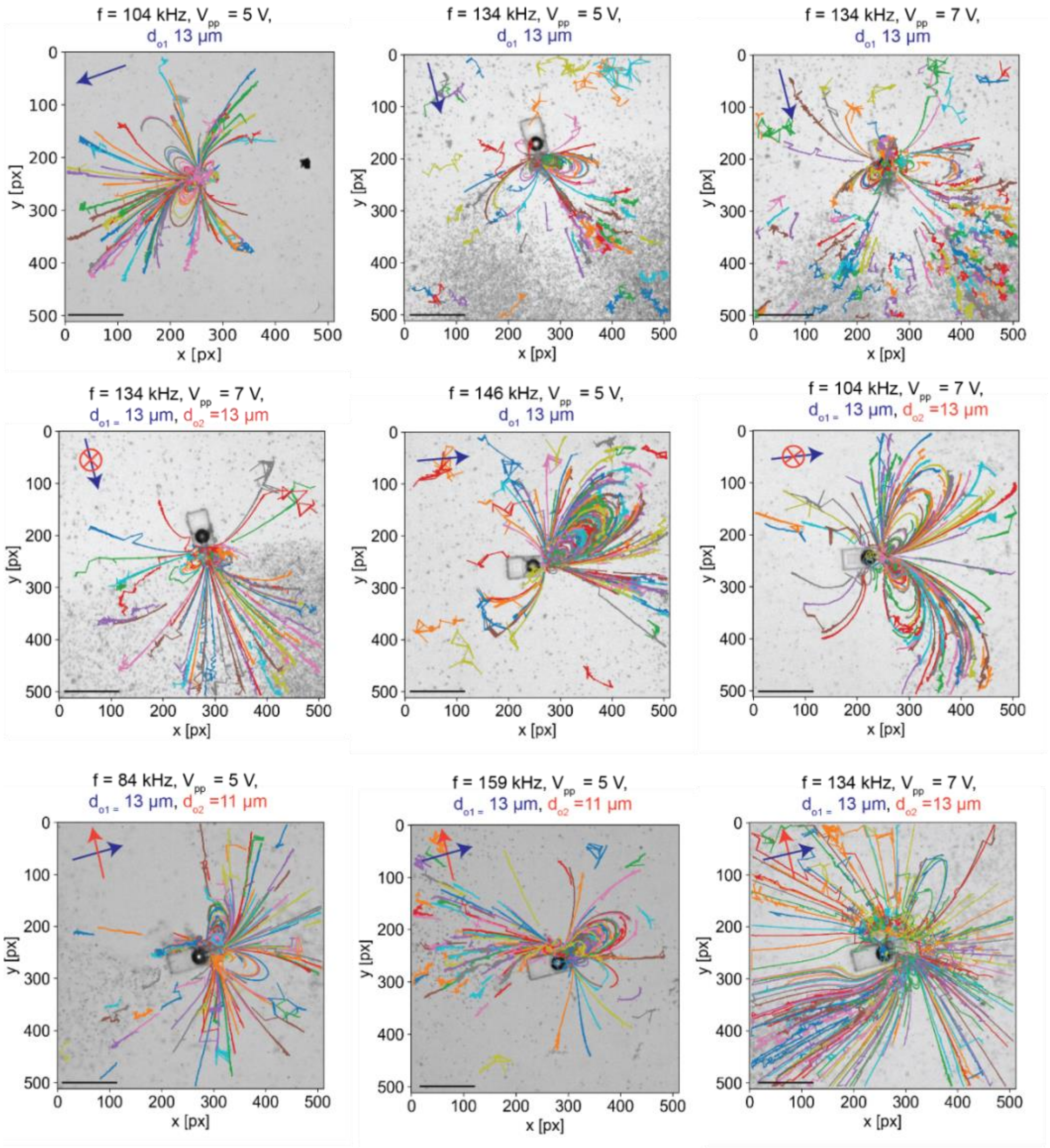

Figure S21. The effect of orifice size on acoustic-streaming. Trajectories of 2-μm-polystyrene particles around an oscillating 30 μm bubble. Arrows show the direction of the orifice. Scale bars: 100 μm.

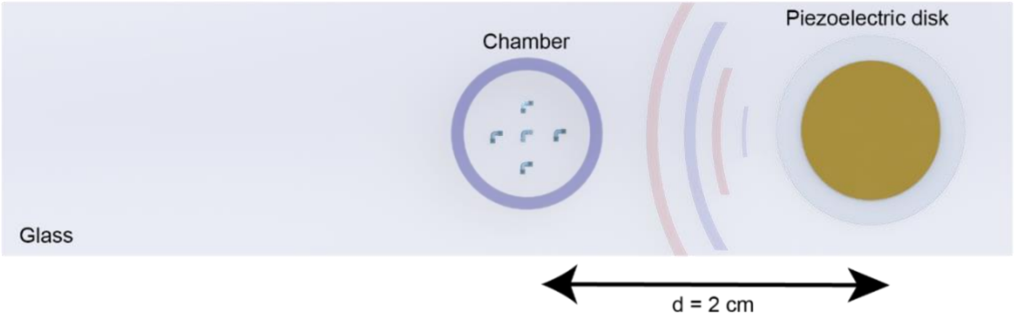

Figure S22. Acoustic setup used in this study with transducer and PDMS chamber. The distance *d* has been set to a fixed value of 2 cm.

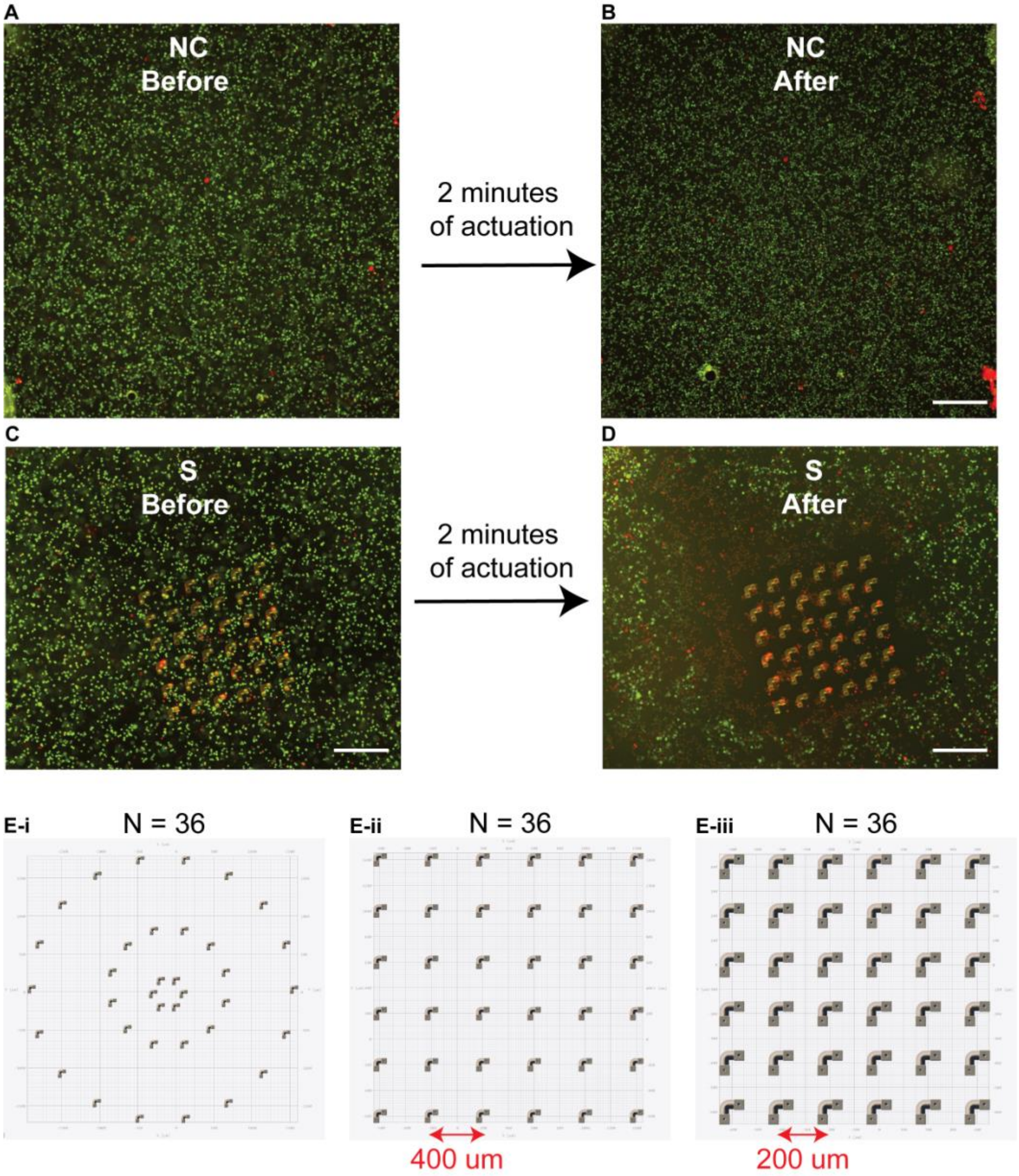

Figure S23. Microscope images of the cell-lysing tests and microrobot arrangements. (**A, B**) Microscope images of the control group experiments. The first image (A) was taken before the actuation and the second image (B) was taken 20 minutes after the actuation. **(C)** Microscopic images of the M test before actuation, and (**D)** 20 minutes after actuation. **(E)** Microrobot arrangements used in this study with rectangular and circular geometry. Scale bars: 500 μm.

Table S2. Cell-lysing test parameters

Chamber diameter	Actuation frequency	Power amplitude	Number of microrobots	Correction factor
6 mm	100 – 150 kHz	7, 9 Vpp	36	4

Supporting Movies

Movie S1. Multimodal locomotion of the acoustically-actuated microrobots. Different locomotion modes are achievable by tuning the acoustic wave intensity and frequency.

Movie S2. Spinning mode of the acoustically-actuated microrobot with 4 orifices—MrSB.

Movie S3. Tunable locomotion modes of the MrSB at different sound wave frequencies and intensities.

Movie S4. The locomotion of the acoustically-actuated microrobots with only bottom orifices – MrB.

Movie S5. Acoustic jet streaming of the interaction of two coupled bubbles with different combinations of orifices.

Movie S6. Cancerous cell-lysing of the anchored microrobots at high flow rates and cell morphological alternations.

Movie S7. Microparticle trapping, transport, and release.

Movie S8. Mixing red and green dye using MrSB at different sound wave frequencies and intensities.

Movie S9. Long-term bubble active stability tests under continuous acoustic actuation.

Movie S10. Locomotion characterization and repeatability tests.

References

[1] T. G. Leighton, *The acoustic bubble*, Elsevier, **1994**.
[2] L. A. Crum, *J. Acoust. Soc. Am.* **1983**, *73*, 116.
[3] P. Zhang, H. Bachman, A. Ozcelik, T. J. Huang, *Annu. Rev. Anal. Chem.* **2020**, *13*, 17.
[4] J.-F. Louf, N. Bertin, B. Dollet, O. Stephan, P. Marmottant, *Adv. Mater. Interfaces* **2018**, *5*, 1800425.
[5] T. A. Spelman, E. Lauga, *J. Eng. Math.* **2017**, *105*, 31.
[6] N. Bertin, T. A. Spelman, O. Stephan, L. Gredy, M. Bouriau, E. Lauga, P. Marmottant, *Phys. Rev. Appl.* **2015**, *4*, 064012.
[7] T. G. Leighton, A. J. Walton, M. J. W. Pickworth, *Eur. J. Phys.* **1990**, *11*, 47.
[8] L. A. Crum, *J. Acoust. Soc. Am.* **1975**, *57*, 1363.
